# Prediction of Kinetic Product Ratios: Investigation of a Dynamically Controlled Case

**DOI:** 10.1021/acs.jpca.2c08301

**Published:** 2023-01-03

**Authors:** Barry K. Carpenter

**Affiliations:** School of Chemistry, Cardiff University, Main Building, Park Place, CardiffCF10 3AT, U. K.

## Abstract

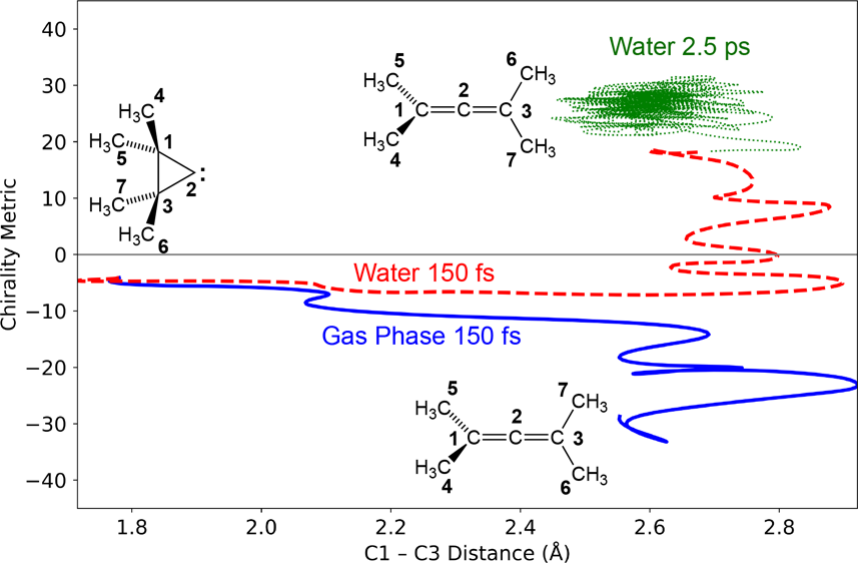

Of the various factors influencing kinetically controlled product ratios, the role of nonstatistical dynamics is arguably the least well understood. In this paper, reactions were chosen in which dynamics played a dominant role in product selection, by design. Specifically, the reactions studied were the ring openings of cyclopropylidene to allene and tetramethylcyclopropylidene to tetramethylallene (2,4-dimethylpenta-2,3-diene). Both reactions have intrinsic reaction coordinates that bifurcate symmetrically, leading to products that are enantiomeric once the atoms are uniquely labeled. The question addressed in the study was whether the outcomes—that is, which product well on the potential energy surface was selected—could be predicted from their initial conditions for individual trajectories in quasiclassical dynamics simulations. Hybrid potentials were developed based on cooperative interaction between molecular mechanics and artificial neural networks, trained against data from electronic structure calculations. These potentials allowed simulations of both gas-phase and condensed-phase reactions. The outcome was that, for both reactions, prediction of initial selection of product wells could be made with >95% success from initial conditions of the trajectories in the gas phase. However, when trajectories were run for longer, looking for “final” products for each trajectory, the predictability dropped off dramatically. In the gas-phase simulations, this drop off was caused by trajectories hopping between product wells on the potential energy surface. That behavior could be suppressed in condensed phases, but then new uncertainty was introduced because the intermolecular interactions between solute and bath, necessary to permit intermolecular energy transfer and cooling of the hot initial products, often led to perturbations of the initial directions of trajectories on the potential energy surface. It would consequently appear that a general ability to predict outcomes for reactions in which nonstatistical dynamics dominate remains a challenge even in the age of sophisticated machine-learning capabilities.

## 1 Introduction

The concepts of kinetically controlled and thermodynamically controlled reactions are familiar to most chemists.

The IUPAC definitions of these terms are as follows:^1^

Kinetic control (of product composition):

The term characterizes conditions (including reaction times) that lead to reaction products in a proportion governed by the relative rates of the parallel (forward) reactions in which the products are formed, rather than by the respective overall equilibrium constants.

Thermodynamic control (of product composition):

The term characterizes conditions that lead to reaction products in a proportion governed by the equilibrium constant for their interconversion and/or for the interconversion of reaction intermediates formed in or after the rate-limiting step. (Some workers prefer to describe this phenomenon as “equilibrium control”.)

Under conditions of kinetic control, three factors have been identified, which influence the “...the relative rates of the parallel (forward) reactions...”. The first is a barrier-height factor, in which parallel reactions leading to the various products face barriers of different free energies. The second is a quantum mechanical factor, in which tunneling of nuclei through potential energy barriers can play a significant role.^2^ The third is a dynamical factor in which the detailed kinematics of the nuclei must be considered.^3^ When the first factor dominates, one can expect classical transition state theory (TST)^4^ to do a good job in predicting product ratios, especially if the location of the transition state is variationally optimized.^5^ When the second factor dominates, classical TST will fail. However, it can be corrected by semiclassical methods, such as the small-curvature approximation,^6^ to include tunneling. The third factor can come into play when the topography of the potential energy surface (PES) is one for which TST is inherently incapable of making predictions, for example, when there are reaction-path bifurcations^7−11^ or when nonstatistical effects such as dynamic matching^12−16^ play significant roles.

Progress is being made on the development of theories for certain aspects of nonstatistical reaction dynamics,^17^ but thus far, there is not an overarching theory that applies to all cases. Instead, one must typically resort to simulation, for example, quasiclassical trajectory calculations.

If one had a theory for the dynamics, it should be able to predict outcomes from initial conditions without need for numerical integration of the equations of motion. It is therefore the issue of predictability that is the focus of the present paper. Developing predictive capability would not be equivalent to deriving a theory, but it should be a useful step in that direction.

The present paper focuses on the question of predictability for trajectories in the special case of reactions with symmetrical bifurcation of the intrinsic reaction coordinate (IRC).^18^ This choice may seem strange because the macroscopic prediction of a product ratio for such reactions is trivial: it will be 1:1 by symmetry, provided that initial conditions have been properly sampled from a canonical or microcanonical distribution. However, there remains a question of microscopic predictability: do the initial conditions for each individual trajectory reliably predict which product it will give? And is it sufficient merely to determine which branch of the bifurcation is initially selected in order to answer that question? By focusing on the symmetrical case, one can be certain that any answers highlight features of the dynamics that are not influenced by differences in gradient leading to the two products, as there would be in all unsymmetrical cases.^19−22^

While the present work was in progress, an important paper by Ess’s group was published, which addressed similar questions.^23^ It focused on two reactions: the dimerization of cyclopentadienone and the extrusion of N_2_ from an azo compound to give semibullvalene. These authors studied a variety of machine-learning (ML) models to make predictions about product formation from initial conditions of the trajectories. Their conclusions are compared with those from the present work in the Results and Discussion section of this paper. Other results from this group are also of relevance to the present work.^24,25^

**Figure 1:**
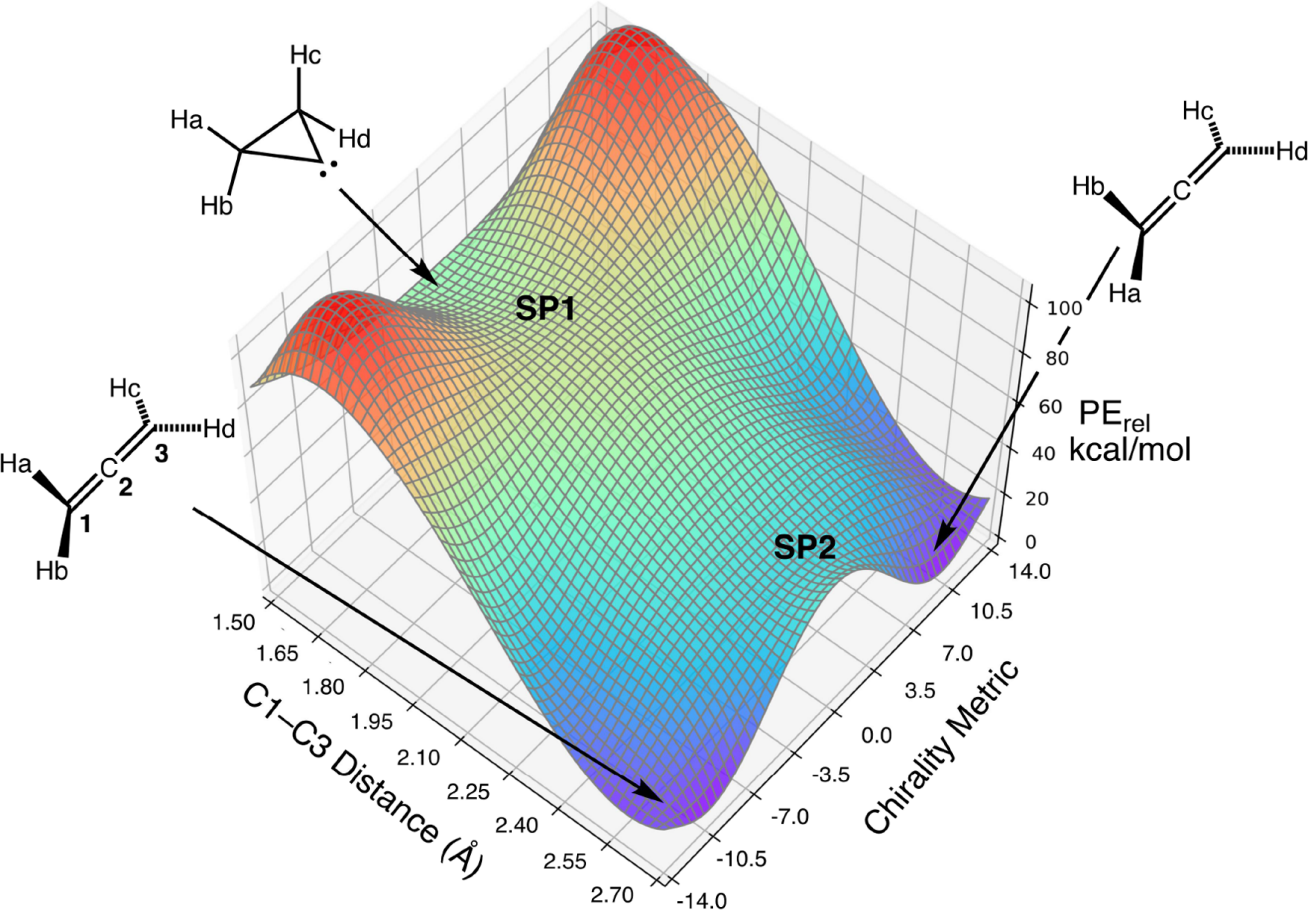
Schematic projection of the PES for ring opening of cyclopropylidene to allene. The carbon numbering is shown on the left-hand allene structure. The chirality metric used to label one of the axes is defined later in the text.

It is useful to introduce the questions addressed in the present work by considering a typical two degree-of-freedom projection of a PES with a symmetrical IRC bifurcation (Figure 1). The reaction is the ring opening of cyclopropylidene to allene, which was one of the first to be shown to have a bifurcating IRC.^26,27^

As is typical for surfaces exhibiting a reaction-path bifurcation, the PES in Figure 1 exhibits two index-one saddle points, labeled SP1 and SP2. As is also typical for surfaces of this kind,^28^ SP2 is lower in potential energy than SP1. Consequently, trajectories initiated from the vicinity of SP1 and integrated at constant total energy are guaranteed to have more than enough energy to surmount SP2. This feature of the PES blurs the distinction between kinetic and thermodynamic control because the reaction conditions might not permit equilibrium among all the species on the PES, but the reaction exothermicity would potentially allow the products to interconvert. Several questions arise from these observations and form the basis for the present paper.

(1) Can the initial conditions for a trajectory initiated in the vicinity of SP1 be used to predict which branch of the bifurcation will be selected?
(2) The full PES for this system has 15 degrees of freedom. Does intramolecular vibrational energy redistribution (IVR) happen sufficiently rapidly for the 13 degrees of freedom not shown in the projection of Figure 1 to act as a bath that can absorb the excess kinetic energy and allow trajectories to stay in the initially accessed product well for extended periods of time?
(3) How does adding complexity to the molecular structure (e.g., by replacing each hydrogen of cyclopropylidene by a methyl group) influence the answers to questions (1) and (2)?
(4) Can intermolecular energy transfer to a solvent bath occur sufficiently rapidly to quench a chemically activated product and cause it to stay in the initially accessed product well?
(5) If the answer to question (4) is yes, does the detailed mechanism of solute to bath energy transfer influence the direction of the trajectory and hence change which product well is initially selected?

## 2 Computational Methodology

### 2.1 Electronic Structure Calculations

The electronic structure programs used for the calculations and their full literature citations are presented in the Supporting Information.

**Figure 2:**
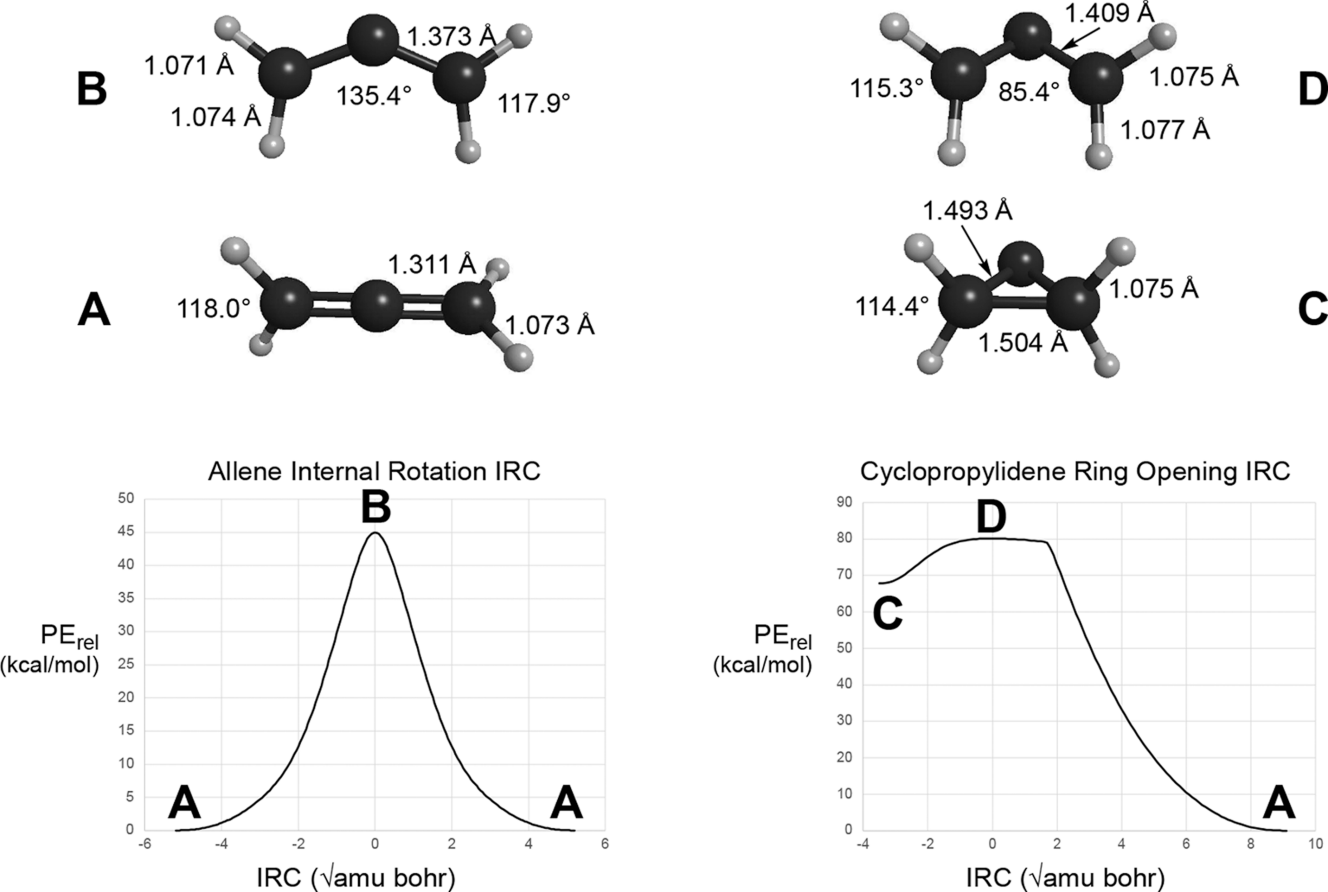
IRCs and stationary-point structures for the ring opening of cyclopropylidene and the internal rotation of allene. All results are at the CASSCF(4,4)/6-311G(3d,2p) level. The upper-case letters beside each structure serve as keys to locate the structure on its corresponding IRC plot. Note that structure **D** corresponds to **SP1** and **B** to **SP2** in Figure 1.

It has been reported that B3LYP density functional theory (DFT) disagrees with complete active space self-consistent field (CASSCF) theory on the symmetry of the PES for ring opening of cyclopropylidene to allene.^29^ CASSCF finds a saddle point of *C*_S_ symmetry with an IRC that bifurcates on the product side of the transition state. The branches of the bifurcation lead to allenes that are enantiomeric if the hydrogens are uniquely labeled (see Figure 1). In contrast, the DFT method finds enantiomeric saddle points of *C*_1_ symmetry for the ring opening, with each being connected to a single product enantiomer. A CCSD(T)/6-31G(d) calculation supported the DFT picture.^29^ The authors ascribed the difference in predictions to the lack of dynamic electron correlation in the CASSCF model. They also argued that multireference effects were negligible, and that their single-reference methods should be reliable. However, as detailed in the Supporting Information, UωB97x-D/cc-pVTZ DFT calculations,^30^ which include both dynamic correlation and dispersion effects, find a *C*_S_-symmetry saddle point. Furthermore, the <*S*^2^> value at the saddle point was found to be 0.2268, indicating that the wave function does, in fact, have modest multireference character. Consequently, it appears that multireference methods are preferable for the PES. For the present work, CASSCF(4,4)/6-311G(3d,2p) calculations were used. The key stationary-point structures and IRCs are shown in Figure 2.

**Figure 3:**
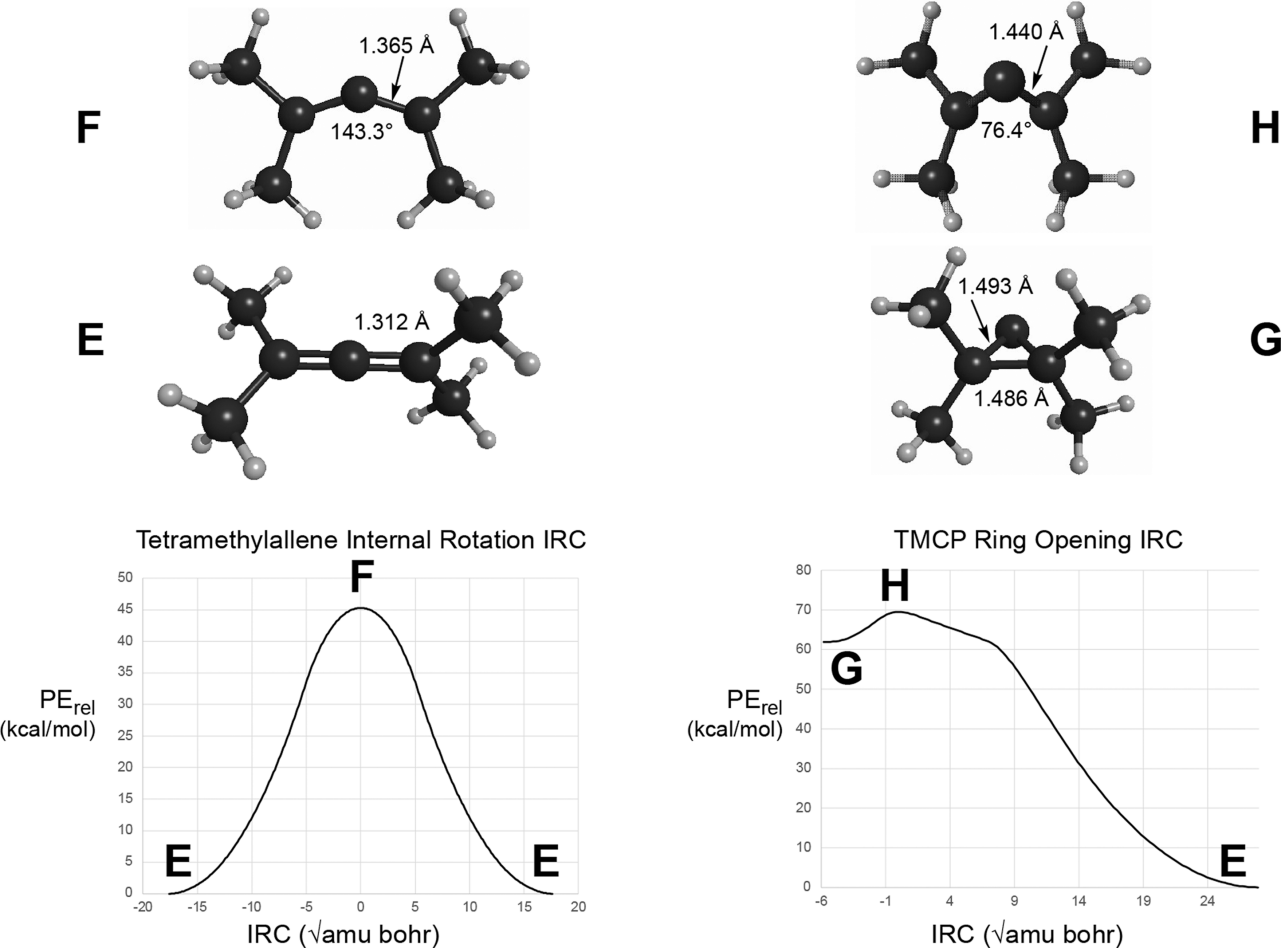
IRCs and stationary-point structures for the ring opening of TMCP and the internal rotation of tetramethylallene. All results are at the UB3LYP-D3/6-31G(d,p) level. The upper-case letters beside each structure serve as keys to locate the structure on its corresponding IRC plot.

In addition to the parent cyclopropylidene, calculations were carried out on its tetramethyl analogue. This time, the B3LYP model did find a *C*_S_ saddle point for its ring opening. The stationary points and IRCs were investigated at the UB3LYP-D3/6-31G(d,p) level. The relatively small basis set was necessary because, in addition to exploring the minimum energy paths, direct-dynamics trajectory calculations were carried out to provide data for training and testing of the artificial neural network (ANN) described in the next section. These were rather time-consuming calculations and were not feasible with a very large basis set, nor with a more sophisticated density functional, such as UωB97x-D. The results of the IRC calculations are summarized in Figure 3. Note that, although the ring-opening saddle point does have *C*_S_ symmetry, the tetramethylcyclopropylidene (TMCP) does not have a *C*_2v_ structure. Apparently, the steric clash between methyl groups on C1 and C3 destabilizes the *C*_2v_ structure and causes a distortion to a chiral, near-*C*_2_ structure. So, for this molecule, there are apparently bifurcations of the IRC on *both* sides of the saddle point. In order to preserve clarity of the diagram, only a few geometrical parameters are shown in Figure 3, but the full molecular structures of the stationary points are provided in the Supporting Information.

### 2.2 Generation of Hybrid Molecular Mechanics—ANN PESs

Although cyclopropylidene and TMCP have few enough atoms to make direct-dynamics trajectory calculations feasible with CASSCF or DFT evaluation of energies and derivatives at each time step, the questions presented in the introduction required that the dynamics also be evaluated in simulations of condensed phases. Those calculations would not currently be feasible with credible electronic structure methods applied to all atoms. One could, instead, try some kind of embedding scheme, such as ONIOM,^31^ but that comes with its own set of challenges.^32^ An alternative is to fit an empirical valence-bond potential to electronic structure calculations for the solute and then to use molecular mechanics for the bath atoms.^33^ However, a potentially more accurate method has recently become available and has been adopted for the present work.

The new method involves training an ANN to reproduce energies and derivatives from the chosen electronic structure method for the solute. That idea, in general, is not new,^34^ but the recent implementation has novel features. All ML approaches to evaluation of PESs face the same problem: the potential must be permutationally invariant for chemically equivalent atoms and must also be invariant with respect to rotation and translation of molecular structures. Taken together, these requirements rule out conventional Cartesian or internal coordinate representations of molecular structures. Several possible solutions to this problem have been proposed.^35−37^ The approach adopted here is that developed by Artrith, Urban, and Ceder (AUC).^38^ They represent local environments of each atom type by radial distribution functions (RDFs) and angular distributions functions (ADFs). The RDFs and ADFs themselves are expressed in basis sets of Chebyshev polynomials. Once an ANN has been trained and tested, it can be used in molecular mechanics packages such as TINKER^39^ for direct-dynamics calculations.

Two small modifications of the original AUC approach have been made for the present study. The original work was aimed principally at description of extended structures for materials-science applications. In that context, it was usually sufficient to have one ANN represent each chemical element because atoms of a given element rarely occurred in very different environments. For the present purposes, it was found that using a single ANN for all the carbons of cyclopropylidene or TMCP did not result in accurate reproduction of electronic structure results. Consequently, ANNs were trained for two different carbon-atom types in cyclopropylidene and three different types in TMCP.

The second modification concerns the incorporation of the ANNs into the TINKER molecular mechanics package. In the original approach,^40^ the ANNs would be designed to do all the work of evaluating potential energy and its derivatives at a given time step of a trajectory calculation. In the present implementation, the MM3 force field has been used in conjunction with the ANN potentials to give a hybrid model, which is here called MM-ANN. The reason for this choice concerns the representation of bath atoms and their interactions with the solute atoms. If one tried to train ANNs for all the atoms in the system, one would be confronted with the unstructured nature of liquids. The training would require a very large number of electronic structure calculations on the solute and bath in various energetically accessible configurations in order to develop an accurate model. The expense of those calculations becomes comparable to that for doing direct-dynamics with the electronic structure model, which is precisely the problem one is trying to avoid. The alternative, adopted here, is to use a molecular-mechanics force field, such as MM3, for the bath atoms and MM3 plus ANN for the solute atoms. In MM3, nonbonded interactions consist of empirical van der Waals, charge–charge, charge–dipole, and dipole–dipole terms.^41^ These apply to both intermolecular and intramolecular nonbonded interactions. If the solute–bath interactions are to be properly represented, the MM3 terms must be retained for the solute. This can be achieved if the collected ANNs are used not as a predictor of energies and derivatives from the electronic structure methods, as the AUC procedure calls for, but instead as a corrector for the MM3 estimates of those quantities.

**Figure 4:**
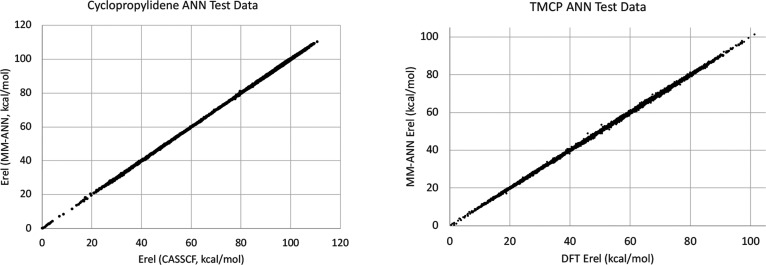
Comparison of MM-ANN potential energies with those from CASSCF (for cyclopropylidene) or DFT (for TMCP) values using the test data sets in each case. For the cyclopropylidene PES, energies were predicted with RMS error of 0.22 kcal/mol. For the TMCP PES, the RMS error was 0.38 kcal/mol.

The structures used for training the ANNs were generated by running direct-dynamics trajectories with quasiclassical normal-mode sampling from a canonical distribution at 298 K. The trajectories were run at the CASSCF level described above for cyclopropylidene and at the DFT level for TMCP. Several hundred trajectories were run for each system, with potential energies and derivatives saved for every time step. Data points from the respective IRC calculations were added to those generated from the trajectories because they provide low PE data points not available from the trajectory data. This procedure resulted in 55,228 data points for the cyclopropylidene ANNs and 82,504 data points for the TMCP ANNs. Details of the ANNs are provided in the Supporting Information. In each case, 10% of the data points were reserved for testing the MM-ANN model after training. The results of the tests are shown in Figure 4.

### 2.3 Analysis of Trajectory Results

The principal questions for each trajectory were which of the enantiomeric product wells it initially encountered and whether it stayed in that well for an extended period of time. For the simulations run in the presence of a bath, there were additional questions about the efficiency of intermolecular energy transfer between the reacting solute and the bath and also about the influence of the bath on the initial choice of product well.

Analysis of the initial direction of each trajectory was facilitated by projection into a suitable 2D space that could depict both the progress of reaction and distinguish between enantiomeric products. The progress of reaction was conveniently monitored by computing the C1–C3 distance at each time step (see Figure 1 for the atom numbering). The distinction between enantiomers was less straightforward. The obvious choice of using a dihedral angle was complicated by two factors. In the case of the cyclopropylidene reaction, an HCCC dihedral becomes ill defined once the CCC angle reaches 180°, as it does in the products. In addition, out-of-plane bending of the methylenes can significantly change that dihedral without influencing the overall chirality of the molecule. The TMCP ring opening suffers from similar issues. Avnir and co-workers have developed a continuous symmetry metric that could solve the problem in principle,^42^ but it involves quite complex calculations and was found to be too slow to apply at each of the time steps of each trajectory.

Eventually, the metric selected was developed from a procedure for identifying enantiomers reported by Cieplak and Wisniewski.^43^ They describe evaluating the sign of determinant χ (eq 1) as an identifier of absolute configuration for four atoms with Cartesian coordinates (*x*_1_, *y*_1_, *z*_1_) ...(*x*_4_, *y*_4_, *z*_4_).
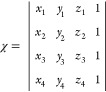
1

**Figure 5:**
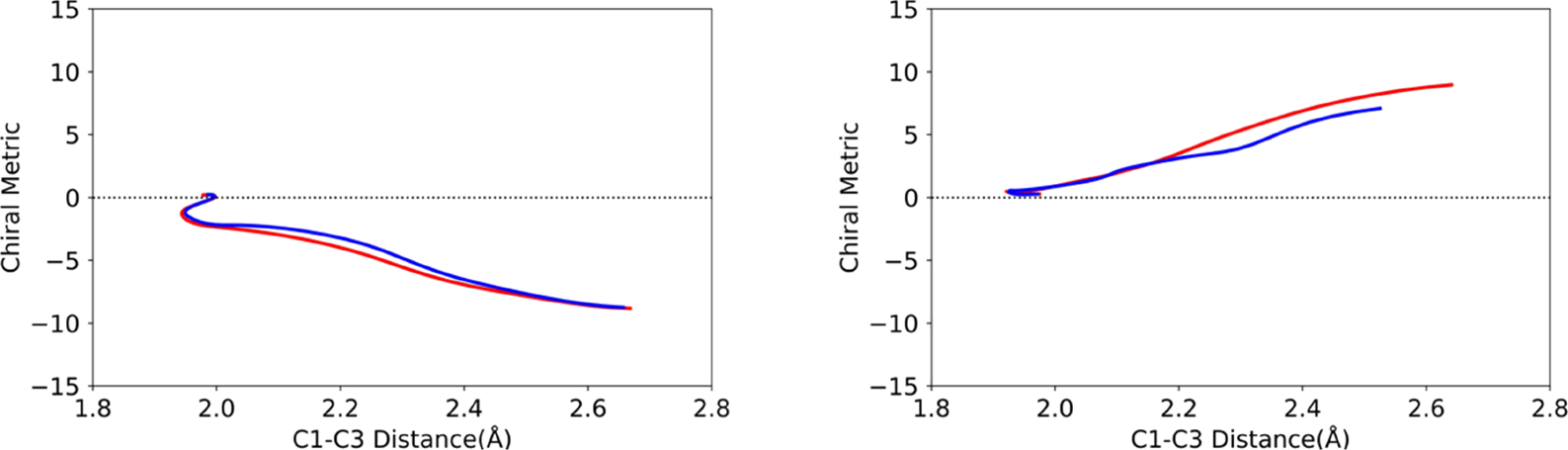
Comparison of CASSCF (red) and MM-ANN (blue) trajectories for cyclopropylidene ring opening with identical initial conditions. Trajectories were propagated for 50 fs in each case. The product enantiomer is defined as M for the left-hand example and P for the right-hand example.

However, it turns out that the value of χ, in addition to its sign, provides a useful continuous metric of chirality. The determinant is invariant with respect to translation and rotation (proofs of these assertions are provided in the Supporting Information) and is very quick to calculate and so, for all the reasons listed, was chosen as the chirality metric for the trajectories. In the case of the cyclopropylidene ring opening, the four atoms were the hydrogens; for TMCP, the methyl carbon atoms were used. Figure 5, which compares direct-dynamics CASSCF and MM-ANN trajectories for cyclopropylidene ring opening, serves to illustrate the use of the chirality metric.

Figure 5 shows that the MM-ANN potential does not exactly match the CASSCF potential against which it was trained, but this is a very rigorous test because small differences in energy or derivatives at any time step of a trajectory will be amplified as the propagation proceeds.

## 3 Results and Discussion

### 3.1 Ring Opening of Cyclopropylidene

#### 3.1.1 Prediction of Product Selection from Initial Conditions in Gas-Phase Simulations

Empirically, it was found that that the cyclopropylidene trajectories, initiated in the vicinity of the SP1 saddle point (see Figure 1), with a sign of the imaginary normal mode eigenvector pointing in the product direction, would reach one or other product well in ≤70 fs. Using the sampling procedure described in Section 2.2, 574 direct-dynamics MM-ANN trajectories were run for 70 fs. Of these, six were found to recross and return to the reactant. The recrossing indicates that, as is commonly the case,^44^ the true transition state for the reaction is not located precisely at the PES saddle point. Of the remaining 568 trajectories, 287 were found to give the M product, meaning that the chirality metric was negative, and 281 the P product, with a positive sign for the chirality metric (see Figure 5). A study was then undertaken to determine whether, for each trajectory, the product selection could have been predicted from the initial conditions. Only one approach to this task was undertaken because, as described below, the first one tested was sufficiently successful that no others seemed necessary.

The approach to prediction was based on a previously described vectorial analysis,^45^ comparing the direction of the reaction-coordinate eigenvector with the vectors connecting the saddle point to one product or the other in mass-weighted Cartesian coordinates. In the original work, this analysis was used to predict macroscopic product ratios, but as described above, that ratio is of no interest in the present case because it must be unity by symmetry. However, one could imagine using a similar approach to make microscopic predictions for individual trajectories. Specifically, for each trajectory, one would approximate the path from the starting structure to either product enantiomer as a linear synchronous transit (LST)^46^ in mass-weighted Cartesian coordinates and then determine the direction of each of those vectors. The preferred product would be taken to be the one whose LST vector was more closely aligned with the direction of the initial mass-weighted Cartesian velocities.

**Figure 6:**
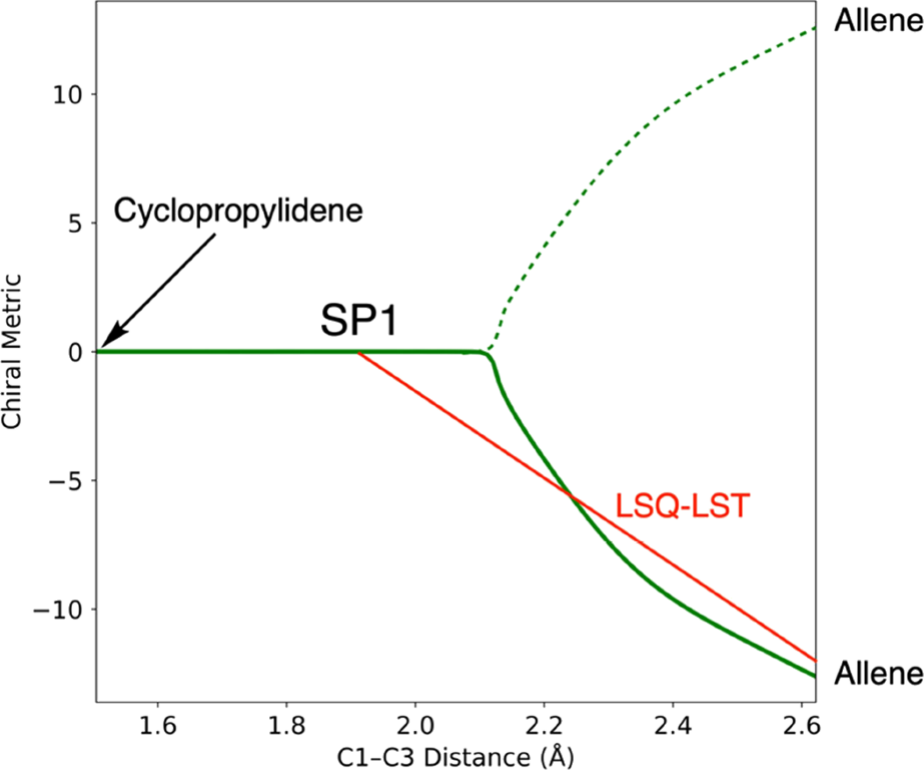
Projection of the cyclopropylidene ring-opening IRC into the 2D space used for trajectory analysis. The calculated IRC is the solid green curve. The dashed green curve is the second branch of the bifurcating IRC, inferred by symmetry. The red line, labeled LSQ-LST, is a schematic representation of the optimum LST approximation to the segment of IRC from SP1 to a product.

Projection of the IRC for the reaction onto the 2D space used for analyzing the trajectories did not provide much encouragement for this approach because, as shown in Figure 6, the IRC was found to be highly nonlinear. The reaction was found to proceed by almost pure C1–C3 stretching well past the SP1 saddle point, but then it suddenly switched to a mixture of torsion and C1–C3 stretching.

As is inevitable, the IRC algorithm arbitrarily picked one branch of the bifurcation. The result is shown as the solid green curve in Figure 6. The second branch was inferred by symmetry and is shown as the dashed green curve. The prediction procedure required that the segment of the IRC from the saddle point SP1 (see Figure 1) to a product be approximated as straight line. The optimum direction of that line was taken to be the least-squares best fit to the IRC segment from SP1 to the product. It is shown schematically as the red line labeled LSQ-LST in Figure 6, but was in reality calculated in the full dimensional space of mass-weighted Cartesian coordinates. The procedure for doing so is described in the Supporting Information. A second LSQ-LST exists for formation of the enantiomeric allene product. The directions of these two vectors were then compared with the direction of initial mass-weighted velocity vector in order to make the product prediction.

Despite the highly nonlinear nature of the IRC, this linear prediction algorithm proved surprisingly effective at choosing the correct product from the initial conditions. Of the 568 trajectories proceeding directly to a product, the linear algorithm correctly predicted the outcome for 547, that is, 96.3%.

#### 3.1.2 Investigation of Long-Time Behavior for the Trajectories in Gas-Phase Simulations

The purpose of running trajectories for a longer time was to find out whether one could determine a “final” outcome for the trajectories because that would have greater experimental relevance than the initial selection of product wells on the PES. However, there is an unavoidable arbitrariness about the choice of duration for the trajectories. First, there is the practical issue of computational cost for running trajectories for a very long time. Second, there is the issue of ergodicity. For systems of low dimensionality, one can sometimes find phase-space barriers that prevent trajectories from accessing all energetically available regions of a PES.^47,48^ However, for high dimensional systems of the kind studied here, there is no evidence to date of such behavior. In other words, it is presumed that the dynamics are ergodic, meaning that constant energy trajectories will traverse all accessible regions of the PES, given sufficient time. Hence, no trajectory will settle in a potential well permanently. Instead, one must ask whether a trajectory stays in a particular well long enough for the outcome to be chemically meaningful. There is no obvious algorithm to tell one how long that should be. In the present work, taking into account the constraint of computational cost, a total duration of 2.5 ps was selected for each trajectory, but other choices could certainly have been made.

**Figure 7:**
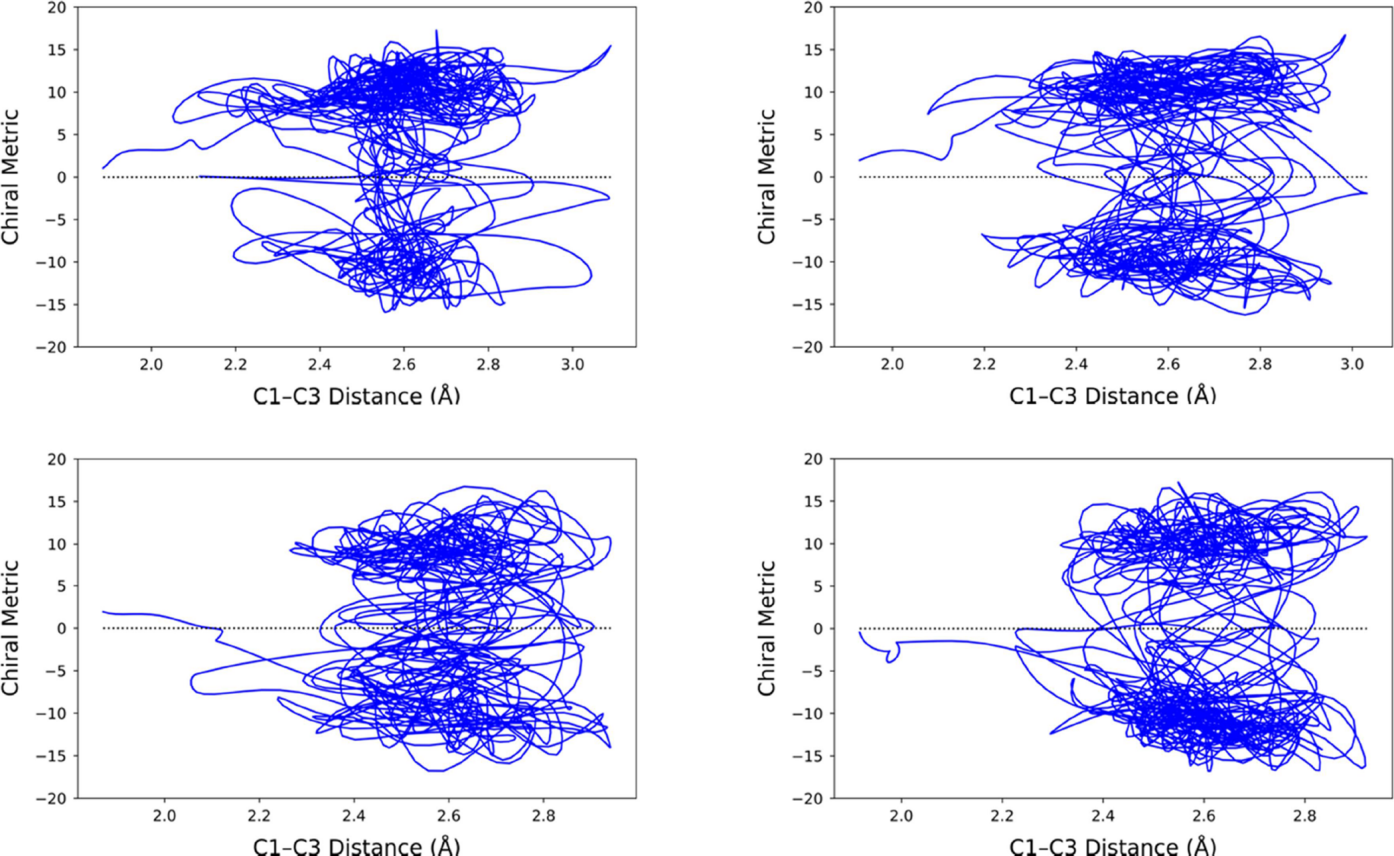
Representative trajectories for the ring opening of cyclopropylidene, run for 2.5 ps in a gas-phase simulation.

Any optimism that one might have had about the apparent success of the linear prediction algorithm was quickly dashed once the trajectories were run for 2.5 ps instead of 70 fs. A few representative outcomes are shown in Figure 7.

Every one of the trajectories looked like this: no matter which product well the trajectory might have accessed initially, it did not stay there, but instead crossed multiple times between the two product wells. So, question (2) posed in the introduction could be answered with an emphatic “No.” IVR did not cause the excess energy of the products to be distributed among all the available degrees of freedom quickly enough to allow any of the trajectories to stay in the product well that it first accessed.

The next question to be addressed was whether the situation might be different for trajectories run in the presence of a bath, which might absorb excess energy and thereby suppress crossing between product wells.

#### 3.1.3 Investigation of the Effects of Various Baths on the Trajectory Behavior

In the introduction, two questions were raised about the effect of a bath on the behavior of the trajectories for ring opening of cyclopropylidene: the first was whether the bath would be able to quench the excess energy of the reacting solute and thereby cause trajectories to stay in the initially accessed well on the PES. The second was whether intermolecular interactions between the reacting solute and the bath would substantially change the directions across the PES taken by the trajectories. The first question is relatively straightforward to answer; the second is less so. Although simulation suggests that chiral solvents can induce optical activity in the products of reactions similar to those studied here,^49^ that outcome is not possible for the present investigations. Because the products in the present case are pseudo-enantiomers, distinguishable only after labeling of the atoms, there cannot be solvent effects on the macroscopic product ratio; it will be 1:1 under all circumstances. Hence, answering the question of whether bath atoms substantially change the direction of trajectories for the reacting solute can be achieved only at the microscopic level, that is, trajectory by trajectory. The following protocol was adopted in order to make microscopic comparisons.

The initial conditions for a cyclopropylidene ring-opening trajectory were recorded. The trajectory was then run for the chosen duration in the absence of any bath. The Cartesian coordinates and conjugate momenta were recorded at each time step. The cyclopropylidene at its initial geometry was then embedded in a cubic, periodic-boundary box of the desired bath atoms or molecules. A constant volume and temperature trajectory was run for 10 ps, with the cyclopropylidene atoms frozen. The temperature was maintained at 298 K by a Bussi–Parrinello thermostat.^50^ The volume was defined by the boundary-box dimensions and selected to give the desired 298 K density of the bath. This initial trajectory allowed the bath to equilibrate around the frozen solute. At the end of 10 ps, the cyclopropylidene atoms were given the momenta recorded from the original conditions and their positional constraints removed. Finally, a constant energy and volume trajectory was run on the whole ensemble, for a total of 2.5 ps. Again, the Cartesian coordinates and conjugate momenta of each time step were recorded.

**Figure 8:**
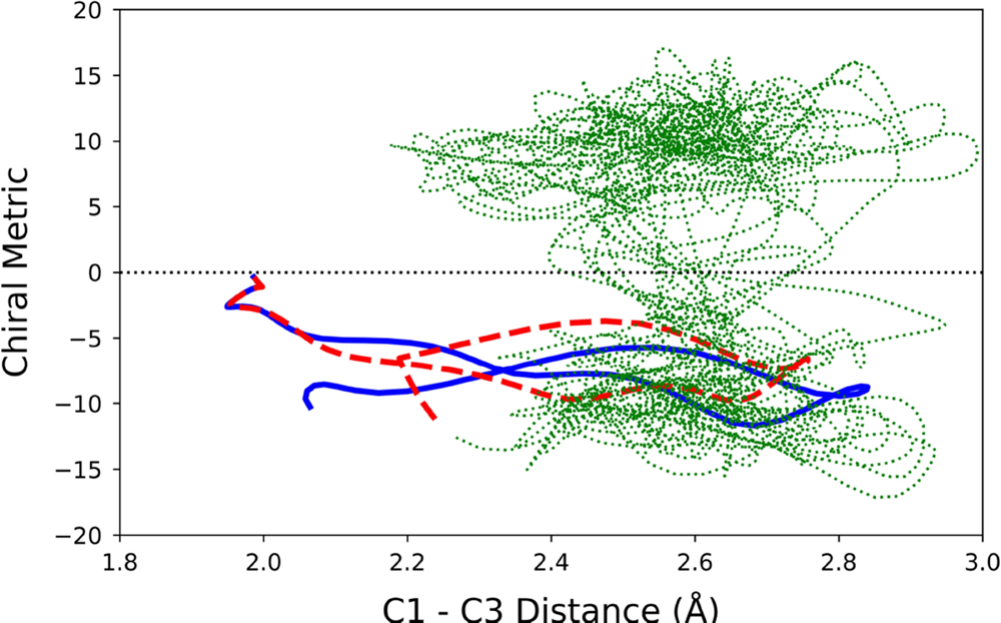
Trajectory for the ring opening of cyclopropylidene in the gas phase (solid blue line, representing 70 fs) and in a bath of helium atoms (dashed red line for 70 fs, followed by dotted green line up to 2.5 ps).

Three baths were investigated. The first was xenon at a density corresponding to the liquid at 298 K.^51^ The second was helium at the same number density as the xenon. This choice was made so that the effects of changing the mass and collisional cross-sections of the bath atoms could be investigated. The third bath was liquid water. An example output, projected into the 2D space described above, is shown in Figure 8. The first 70 fs of the gas-phase trajectory is shown in the solid blue line. The first 70 fs for the trajectory in the presence of the bath, in this case helium, is shown as the dashed red line. The remainder of the trajectory in the bath, up to a total of 2.5 ps, is shown as the dotted green line. The purpose of the color coding is to allow quick comparison of the first 70 fs of the trajectory in the presence and absence of the bath in order to determine whether the bath has significantly changed the initial direction of the trajectory. The green line allows one to see whether the trajectory settles in one product well or continues to switch between the two, as the gas-phase trajectories all do.

**Figure 9:**
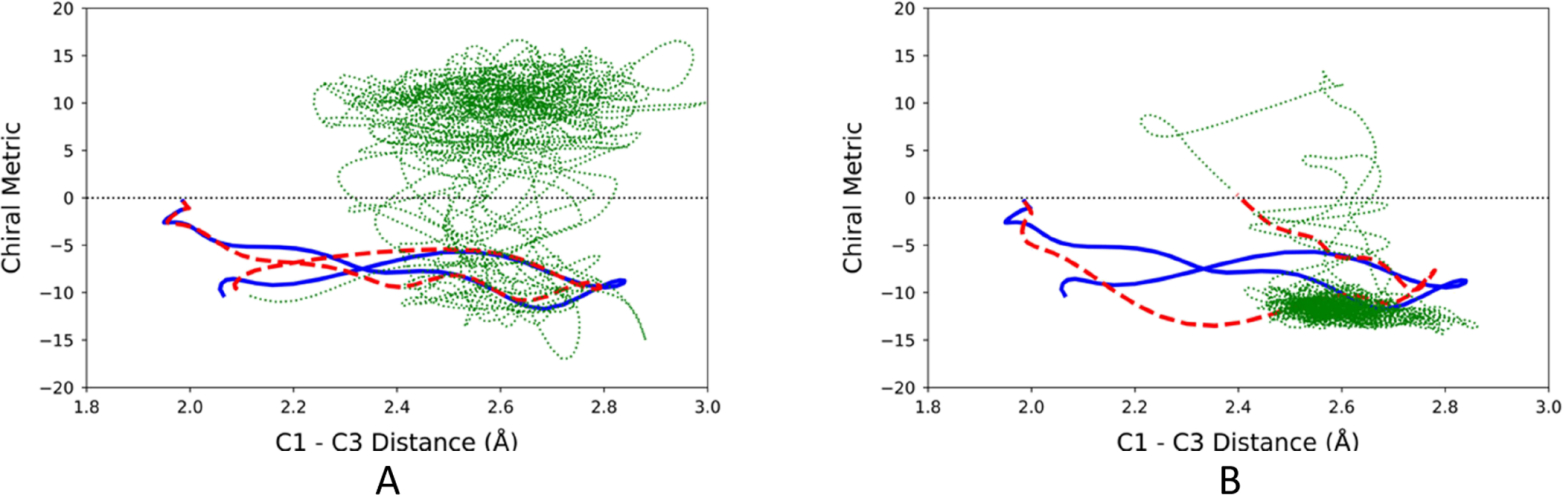
Trajectories for ring opening of cyclopropylidene in liquid xenon (A) and liquid water (B). See caption of Figure 8 for color coding.

What one sees in Figure 8 is that the trajectories in the presence and the absence of the He bath initially follow very similar tracks, as one would expect given that the cyclopropylidene atoms had identical initial conditions. However, very quickly the blue and dashed red lines begin to separate. Unsurprisingly, this separation only increases with time. The green trace shows that, for this trajectory, the He bath has not been successful in quenching the excess energy of the reacting molecule sufficiently rapidly to prevent the trajectory switching between product wells. In total, 574 trajectories were run in the He bath, and in none of them did the trajectory settle in the initially accessed product well. Trajectories were then run in the simulated liquid xenon and liquid water baths. Examples of both are shown in Figure 9. For ease of comparison, the same initial conditions for the solute atoms were used in all the trajectories depicted in Figures 8 and 9.

**Figure 10:**
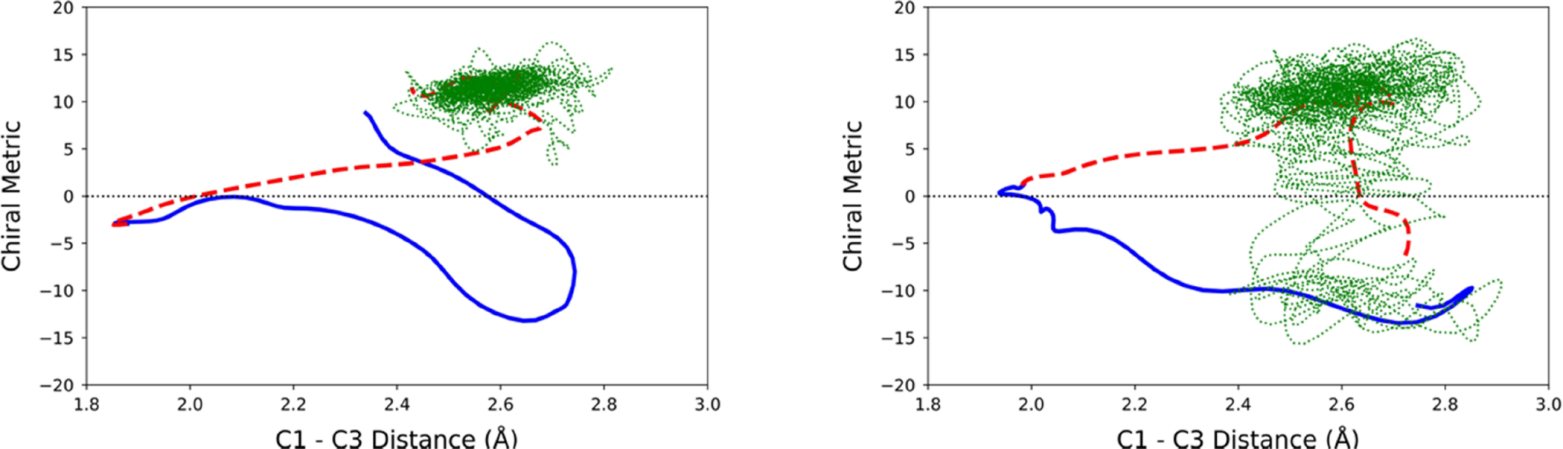
Trajectories for the ring opening of cyclopropylidene in water. See caption of Figure 8 for color coding.

For the particular trajectories illustrated in Figures 8 and 9, one sees that the Xe bath is not very different in its effect from the He bath. If anything, the condensed-phase trajectory deviates less from the gas-phase one in Xe than in He. By contrast, the trajectory in liquid water looks very different. After 70 fs, the gas-phase trajectory and the two noble-gas bath trajectories are all comfortably on the negative side of the chirality metric, whereas the trajectory in liquid water is in the process of crossing to the positive side, which indeed it does in the next few fs. The trajectory in water does seem eventually to settle in the M product well, but not before crossing the barrier several times between P and M products. The implication is that water has been significantly more effective than He or Xe in absorbing excess energy from the reacting solute. Examination of the 574 trajectories in each bath supported this conclusion. It is not a surprising outcome because the noble-gas baths can only cool off the solute by vibration-to-translation energy transfer, which is notoriously inefficient, whereas in water, the much more efficient vibration-to-vibration energy transfer is possible.^52^ Despite this higher cooling efficiency for water, in few of the trajectories run in that bath did the reactant stay in the well that it initially accessed. Furthermore, in several cases, as illustrated in Figure 10, the initially accessed well was different between gas-phase and water trajectories.

Further insight into the nature of the interaction between the reacting cyclopropylidene and each of the baths could be acquired by partitioning of the kinetic and potential energy terms at each time step of the trajectories. The MM-ANN Hamiltonian can be written as eq 2:
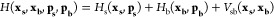
2
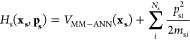
3
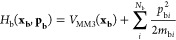
4where **x_s_** and **x_b_** are, respectively, the vectors of Cartesian coordinates for the solute and the bath, while **p_s_** and **p_b_** are their conjugate momenta. In eq 2, the full Hamiltonian is partitioned into a solute-only term, a bath-only term, and a term describing the interaction between solute and bath. The last of these terms depends only on the positions of the atoms, not their momenta. In eq 3, *N*_s_ is the number of atoms in the solute, *p*_s*i*_ is the momentum of solute atom *i*, and *m*_s*i*_ is its mass. The first term on the right-hand side of eq 3 describes the potential energy of the solute, as calculated with the hybrid MM-ANN potential, and the second term describes its kinetic energy. Eq 4 describes the corresponding terms for the bath, except that now the potential energy comes from pure MM3 molecular mechanics. The third term of eq 2 also comes solely from MM3.

**Figure 11:**
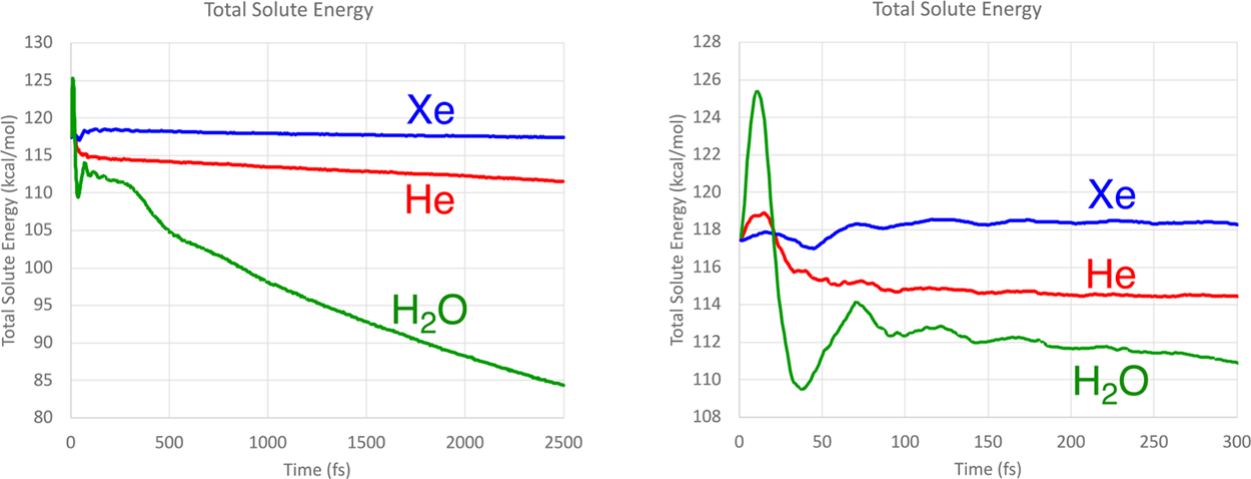
Plots of the quantity *H*_s_(**x_s_,p_s_**) (see eq 3) as a function of time for the ring opening of cyclopropylidene in the three baths He, Xe, and H_2_O. The plots depict averages over 500 trajectories for each bath. The right-hand plot is a scale expansion of the first 300 fs from the left-hand plot.

Because all of the trajectories are run at constant total energy, *H*(**x_s_,x_b_,p_s_,p_b_**) is constant. In the gas phase, all the terms in eqs 2–4 involving bath atoms go to zero, and so *H*_s_(**x_s_,p_s_**) is also constant. However, for trajectories run in the presence of a bath, it is not. Tracking the magnitude of *H*_s_(**x_s_,p_s_**) throughout the course of a trajectory provides information about how energy is exchanged between solute and bath. For a single trajectory, this is a very sharply changing function as, for example, stretching C–H bonds brings solute hydrogens closer to or further away from nearby bath atoms. However, averaging the results over several hundred trajectories smooths out these short-term effects and reveals underlying trends in energy transfer. Figure 11 reveals the result for cyclopropylidene ring opening in the three baths, He, Xe, and H_2_O.

From the left-hand panel of Figure 11, the first obvious conclusion is that water is much more effective than either of the noble gasses for cooling off the reacting cyclopropylidene. Somewhat surprisingly, helium is found to be a slightly more effective quencher than xenon. Troe and co-workers found the same phenomenon in their simulation of the interaction of vibrationally excited benzene and hexafluorobenzene with noble gases, although this seems to be an error in the models for intermolecular interactions because the experimental trend is in the opposite direction.^53^ The scale expansion in the right-hand plot of Figure 11 reveals interesting short-time phenomena that differ substantially among the three baths. The Xe and H_2_O plots exhibit damped oscillations, with the effect being much more dramatic for the water case. By contrast, the He plot shows a single transient, followed by a rapid energy drop, which dies away within about 50 fs.

Examinations of animations of the trajectories revealed the origins of these effects. All arose from the same fundamental phenomenon, which is a change in shape of the solute as the ring opening occurs. For example, the distance between the two carbons of the breaking bond increases by 33% between the saddle point structure and the product. During the equilibration step of the trajectory calculation, the bath was allowed to find an optimum cavity size to house the solute. However, the optimum size for the saddle point is too small for the product. As a result, the solute atoms encounter clashes with nearby bath atoms in the early stages of the ring opening, on the downhill slope of the solute potential as it heads toward the products. These clashes cause the ring opening to reverse, which relieves the intermolecular interactions but raises the potential energy of the solute, as it climbs back toward the saddle point structure. In the meantime, the bath has responded by rearranging the atoms to create a larger cavity. This allows the reaction to proceed in the forward direction once more but may require a few smaller reverses before the cavity is finally large enough to accommodate the product. In xenon, the readjustment of the bath is relatively easy, and so the oscillations are of small amplitude. The situation is different in liquid water. Because of the hydrogen bonding network in water, accommodating a larger cavity for the solute is much more difficult, and so the bath is more resistant to the reaction, leading to the large amplitude oscillations. The helium bath is different again. Because of the smaller collisional cross section and lower mass, helium atoms offer less resistance to the changing solute than xenon atoms do. In fact, there are enough void spaces in the helium bath that it is possible for one or two helium atoms to pick up substantial translational velocity for a short time, as a consequence of being swatted away by the expanding solute. They carry away the approximately 2.5 kcal/mol energy that one can see being lost in the early stages of the reaction.

The left-hand plot of Figure 11 reveals that, after 2.5 ps, the He and Xe baths have absorbed <5 kcal/mol of the approximately 35 kcal/mol excess energy (i.e., energy above the barrier between product wells) in the reacting solute. It is unsurprising, therefore, that these baths are ineffective in causing the trajectories to settle in the first-encountered well. The water bath is much more effective, carrying away an average of 33 kcal/mol after 2.5 ps. However, even this is not rapid enough because trajectories can cross between product wells in <100 fs in the early stages of the reaction.

The conclusion for the cyclopropylidene ring opening is that none of the baths studied is effective at allowing a reactive trajectory to settle in the first PES well that it encounters. As a consequence, having the ability to predict which well a given trajectory will first fall into has no real value.

If intermolecular energy transfer cannot accomplish the desired goal, one may wonder whether IVR might be more successful. The simulations revealed that for cyclopropylidene, IVR was not effective, but increasing the number and nature of the available intramolecular bath modes might improve the situation. It has been known for some time that methyl rotors are particularly good at promoting IVR,^54,55^ and so studies were carried out on a tetramethyl analogue of cyclopropylidene. They are described next.

### 3.2 Ring Opening of TMCP

#### 3.2.1 Prediction of Product Selection from Initial Conditions in Gas-Phase Simulations

Question 3, raised in the Introduction, concerns the ability to predict initial product selection as molecular complexity increases. Would, for example, the simple linear algorithm described in Section 3.1.1, which successfully predicted initial product choices for a molecule of 7 atoms, still be successful with 19 atoms, which TMCP has? Furthermore, should all 19 atoms be included in the analysis for TMCP, or is the information primarily embedded in the positions and momenta of the 7 carbon atoms? The process of finding answers to those questions began by running 1246 trajectories for the ring opening of TMCP in the gas phase. The ring opening was found to be notably slower for TMCP than for cyclopropylidene, taking an average of 150 fs instead of 70 fs. Of these trajectories, 115 were found to recross and return to the reactant. This is a higher percentage than seen for cyclopropylidene (9% rather than 1%), indicating that the true TS is further removed from the saddle point for TMCP than for cyclopropylidene. When the 1131 trajectories proceeding directly to a product were submitted to the linear algorithm described in Section 3.1.1, the results were disappointing. If only the 7 carbon atoms were included, the success rate was found to be 76%. With all 19 included, it was only marginally better at 78%. These may sound reasonably acceptable results, but it must be remembered that one would get 50% correct with a random number generator.

Because the simplest predictive model was not very successful for TMCP, more sophisticated models were investigated. In particular, ML models were tried. The ML algorithms did not readily allow incorporation of the Kabsch rotation, which forms part of the linear algorithm (see Supporting Information), and so the decision was made to convert from a Cartesian basis to an internal coordinate basis for both initial positions and momenta of the TMCP atoms. This was done by computing the Wilson B matrix,^56^ whose elements are given by eq 5.
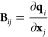
5where **q** is the vector of internal coordinates and **x** is the vector of Cartesian coordinates. The matrix **B** has dimensions 3*N*_s_-6 × 3*N*_s_, where *N*_s_ is the number of atoms of the solute (19 for TMCP). The conversion of Cartesian momenta to their internal-coordinate equivalents was accomplished by premultiplying the vector of Cartesian momenta by the matrix **B**.

1000 of the 1246 trajectories were used to train each model, with the remaining 246 used as test data. Three possible outcomes for each trajectory: M, P, or R were identified. The M and P outcomes correspond to the two enantiomers of the tetramethylallene product, while the R outcome means that the trajectory recrossed to the reactant. Three ML models were tested: random forest and support vector models were found to be little better than the linear model in predicting outcomes from initial conditions. However, a boosted tree model, described in more detail in the Supporting Information, was found to be highly successful. It could predict the outcome (including recrossing) with 98.0% success on the test data and 98.1% on the training data.

#### 3.2.2 Investigation of Long-Time Behavior for the Trajectories in Gas-Phase Simulations

**Figure 12:**
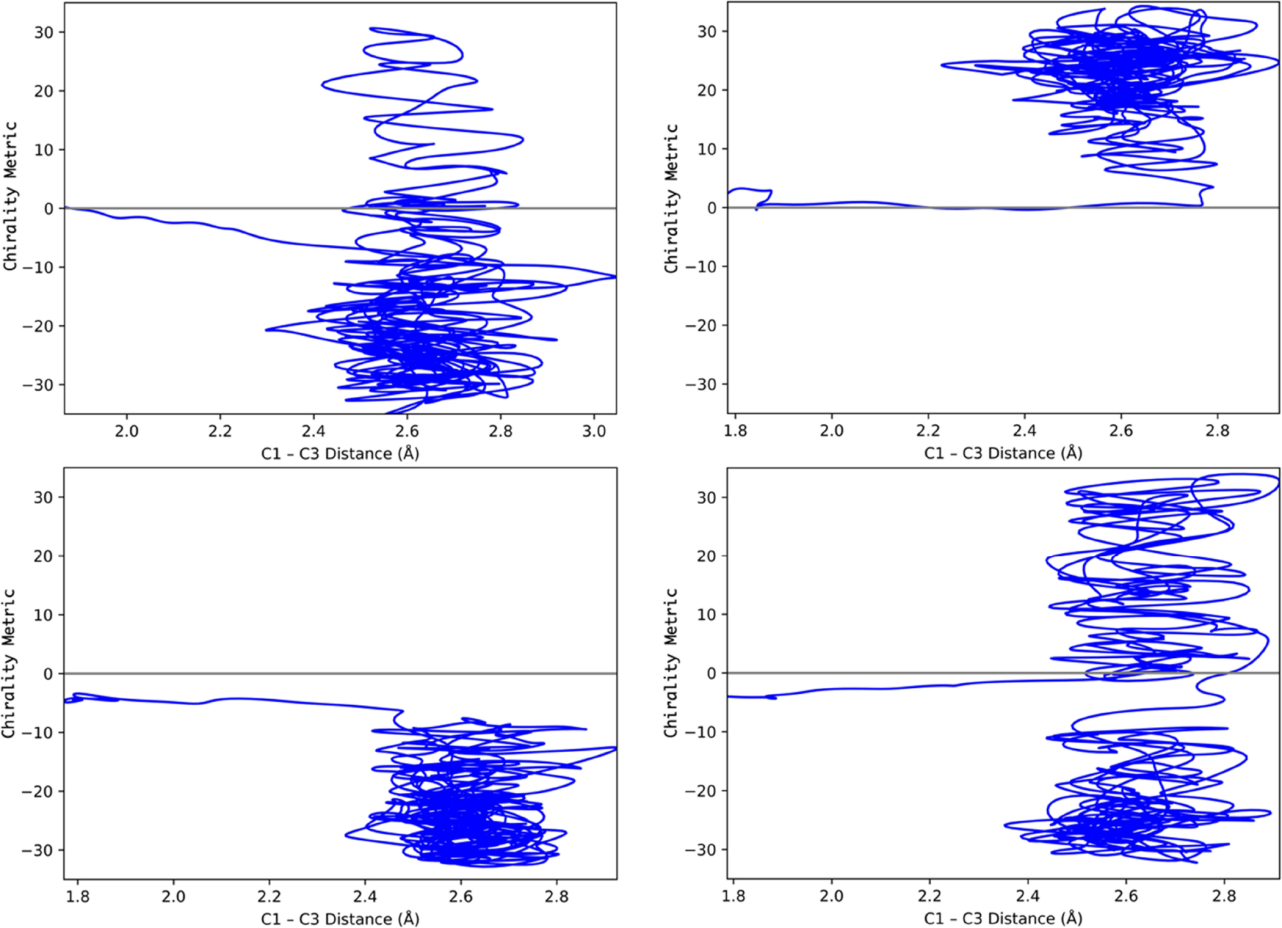
Representative 2.5 ps trajectories for the ring opening of TMCP in the gas phase.

With a successful predictive model for initial product selection established, the question again turned to its value, or otherwise, for predicting final product outcomes. The hope was that, with the extra vibrational states made available by addition of the four methyl groups, IVR might be sufficiently rapid to suppress the switching of trajectories between product wells. As before, this issue was initially addressed by running the gas-phase trajectories for 2.5 ps. Representative results are shown in Figure 12. Now, for the first time, some trajectories were indeed found to settle in the initially accessed product well. However, there were not enough of them to make prediction of final product outcomes useful. Of the 1131 trajectories that progressed directly to a product well, 510 underwent at least one crossing between wells within the 2.5 ps time period. Nonetheless, the outcome was sufficiently encouraging to investigate whether the combined effects of IVR and intermolecular energy transfer to a bath might finally be sufficient to suppress the product switching completely.

#### 3.2.3 Investigation of the Effects of Various Baths on the Trajectory Behavior

**Figure 13:**
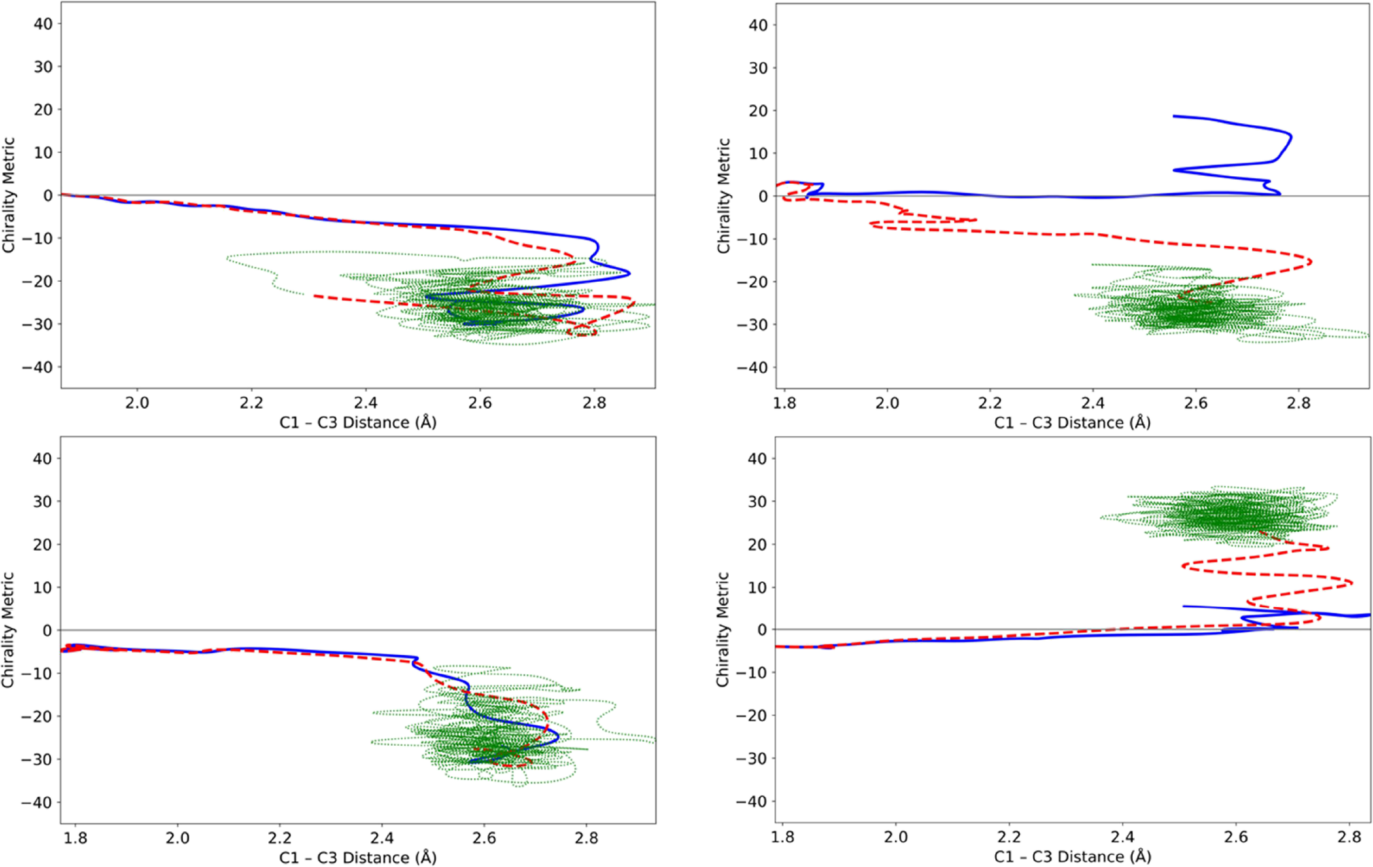
Four trajectories for the ring opening of TMCP in the gas phase (solid blue line, representing 150 fs) and in a bath of helium atoms (dashed red line for 150 fs, followed by dotted green line up to 2.5 ps). The TMCP initial conditions for these trajectories were identical to those for the corresponding trajectories shown in Figure 12.

The procedure adopted for the simulation of condensed-phase trajectories was entirely analogous to that described in Section 3.1.3, and so those details are not repeated here. Figure 13 illustrates the effect of the helium bath on the same trajectories illustrated in Figure 12.

One sees that the two trajectories in Figure 12, which had exhibiting switching between product wells, now show that the trajectory has settled in the initially accessed product well. However, not all the 1246 trajectories showed this behavior. Furthermore, the upper right panel in Figure 13 shows a disturbing outcome: the presence of the bath has changed which product well is initially accessed. Consequently, the ML model, which had correctly predicted the initial product choice for the gas-phase simulation now makes an incorrect prediction for the trajectory run with the helium bath.

**Figure 14:**
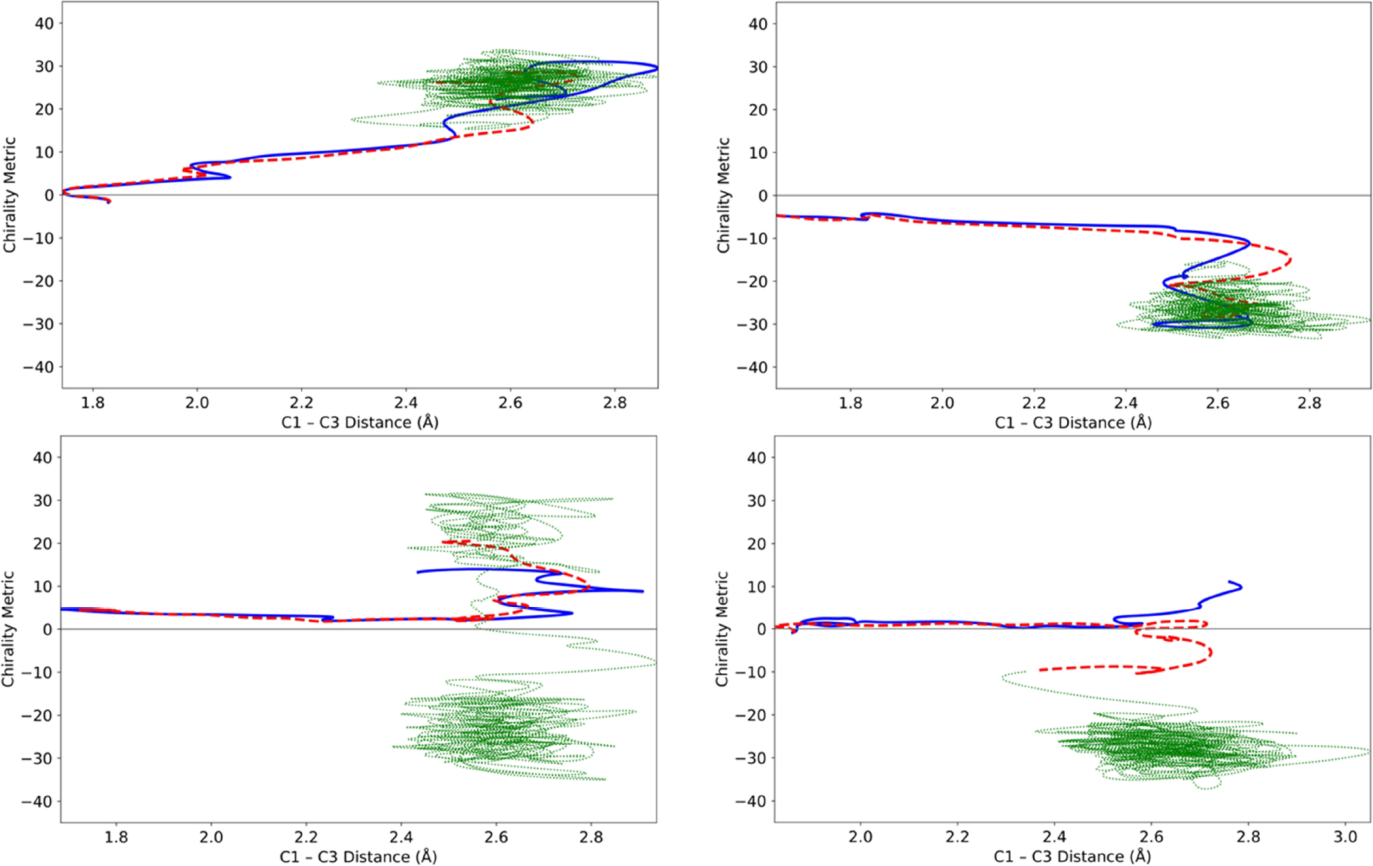
Four trajectories for the ring opening of TMCP in the gas phase (solid blue line, representing 150 fs) and in a bath of liquid xenon (dashed red line for 150 fs, followed by the dotted green line up to 2.5 ps).

**Figure 15:**
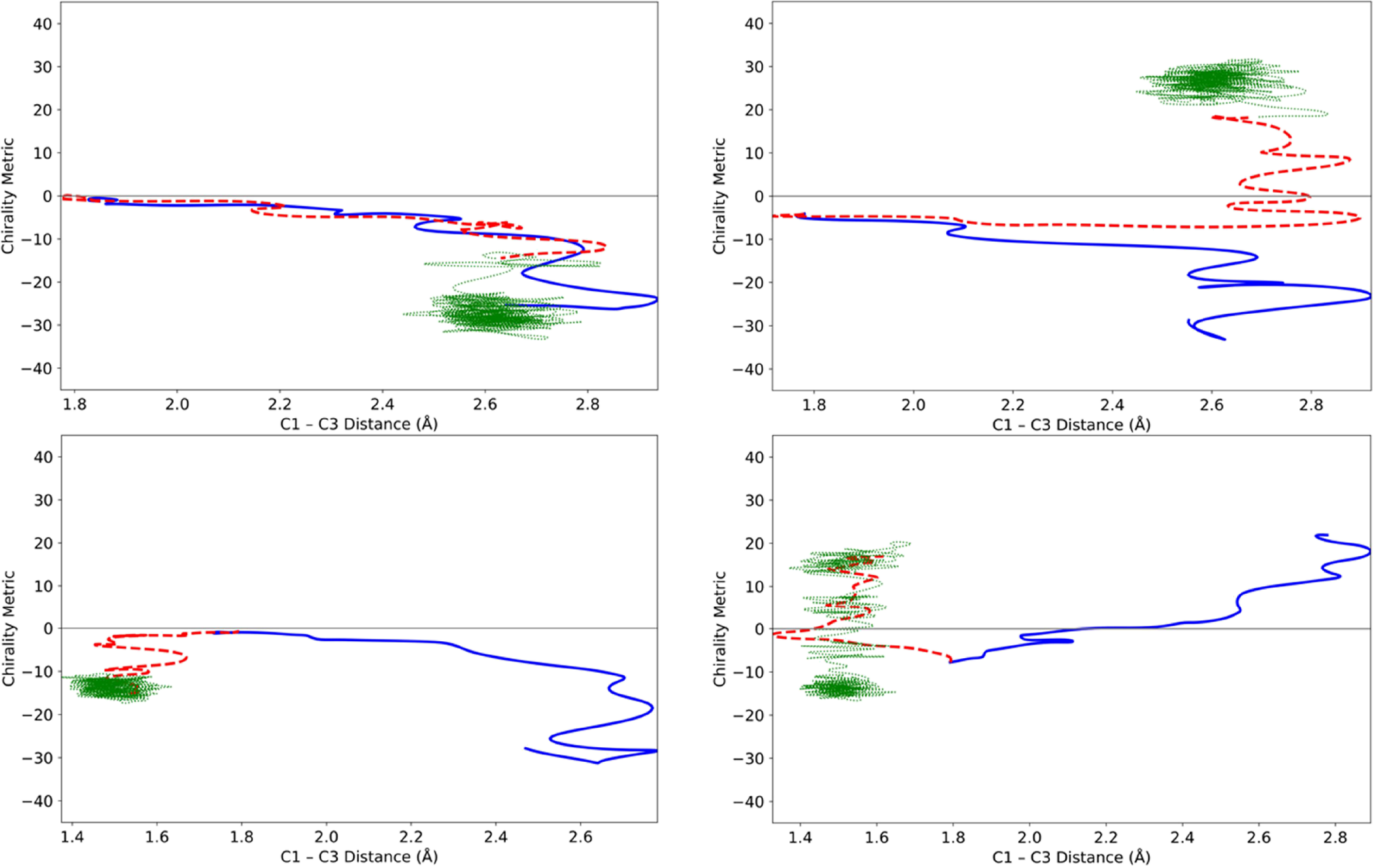
Four trajectories for the ring opening of TMCP in the gas phase (solid blue line, representing 150 fs) and in a bath of liquid water (dashed red line for 150 fs, followed by the dotted green line up to 2.5 ps).

Four trajectories each are shown for the xenon and water baths in Figures 14 and 15, respectively. In the lower two panels of Figure 15, one sees trajectories that proceeded directly to products in the gas phase, but which recrossed to the reactant in water. In the lower left panel, the trajectory has settled in one reactant well, with a negative value of the chirality metric, whereas in the lower right panel, the trajectory switches between reactant wells on either side of the achiral line. The two reactant wells arise because, as described in Section 2.1 and depicted in Figure 3, the DFT calculations found TMCP to have a chiral, near-*C*_2_ rather than an achiral *C*_2v_ minimum-energy geometry. This feature of the PES is correctly reproduced by the MM-ANN potential.

The outcomes for all the trajectories in the gas phase and in the three baths are summarized in Table 1. In the table, the “Recross” category identifies the percentage of trajectories that returned to the reactant, despite being given initial conditions that pointed them in the product direction. The “Product Switch” category identifies how many trajectories crossed the barrier between the two product wells at least once. The numbers in this row provide the clearest indication of the ability of the various baths to quench the excess energy in the initially formed product. The “Wrong Product” category indicates what percentage of the initially formed products in each bath were different from those for the corresponding trajectory in the gas phase. The “ML Success” category shows what fraction of final products would have been correctly predicted by the ML model that was trained on the 150 fs gas-phase results. In order to calculate the numbers in this row, it was assumed that trajectories switching between product wells had an equal probability of eventually settling in one well or the other. The “RN Success” category indicates how successful one would expect to be in predicting outcomes with a random number generator instead of the ML model. The numbers indicated presume prior knowledge of the fraction of trajectories that recross. If one did not make such a presumption, all the “RN Success” entries would be 33.3%.

**Table 1:** Summary of TMCP Trajectory Outcomes for 1246 Initial Conditions in Each of the Baths Indicated^a^

	gas phase (%)	helium bath (%)	xenon bath (%)	water bath (%)
recross	9.2	10.4	9.0	13.7
product switch	45.3	23.0	24.9	6.6
“wrong” product	N/A	26.7	25.2	57.1
ML success	76.9	81.9	80.2	44.9
RN success	42.1	41.2	42.2	39.1

a All trajectories were run for 2.5 ps. See text for the meanings of the various categories.

The following observations can be made about the data in Table 1. The ML model was trained on the 150 fs data in the gas phase, that is, corresponding to the initially chosen minimum (reactant or either product) on the PES. Under none of the conditions summarized in Table 1 did the final outcome match the initial choice with perfect fidelity. As a consequence, the 98% success in predicting initial choices could not be reproduced in the prediction of the final outcomes for each trajectory. The highest success rate among the conditions studied was 81.9% for the helium bath. The IVR rate was not high enough for the “internal bath” corresponding to the non-reactive vibrational modes of TMCP to take up the excess energy sufficiently rapidly to prevent all product switching. Draining excess energy out of the modes that contribute to product switching could be aided by intermolecular energy transfer to an external bath. However, this could not be done in a purely passive fashion: the intermolecular interactions that led to energy transfer necessarily influenced the direction of the trajectories across the PES. The more effective the intermolecular energy transfer was, the greater the perturbation of the trajectory direction. Thus, one sees in Table 1 that water was by far the best energy absorber of the three baths studied, with only 6.6% trajectories leaving the initially selected product well. However, water also had the strongest perturbing influence on the trajectory direction, with 57.1% trajectories leading to a different initial choice of local minimum on the PES from the gas phase result with identical initial conditions for the solute atoms.

## 4 Conclusions

Although classical Hamiltonian dynamics are based on deterministic equations, which would seem to imply perfect predictability of outcomes by definition, in the high-dimensional systems of relevance to chemistry, the dynamics are almost inevitably chaotic. What does that chaos mean for any attempts at practical predictability? Perhaps surprisingly, the answer to that question remains unresolved, in its most general form.^57^ Of the many definitions of chaos, all agree that it is an infinite-time concept.^57^ However, chemical reactions are not infinite-time events; they have a finite duration. Indeed, it is notable that in the IUPAC definition for kinetic control, presented in the introduction to this paper, explicit mention was made of reaction times as a controlling factor. In the laboratory, one might define the duration of a reaction as being the length of time from initiation of the reaction to the isolation of the product(s), which typically occurs on the timescale of hours. In classical dynamics simulations, one might define the duration of a reactive trajectory as the length of time from the crossing of the phase-space dividing surface that represents the transition state to the first access of the product well, in which the trajectory eventually settles. The timescale here is much shorter, typically on the order of picoseconds rather than hours, at least for small molecules. Even if the dynamics of a chemical system are tending toward chaotic behavior in the limit of infinite time, one might still see predictable behavior on a chemically relevant timescale. This paper has concerned numerical experiments on particular reactions—the ring opening of cyclopropylidene and its tetramethyl analog, TMCP—to explore how predictable outcomes are on different timescales and under different conditions.

In short, the findings were that on very short timescales ∼70 fs for cyclopropylidene and ∼150 fs for TMCP, predictability could be very high, >95%. However, these timescales are not really chemically relevant because they do not correspond in any meaningful sense to having seen the reaction to its conclusion. If one observes the dynamics for longer—a duration of 2.5 ps was used in the present work—predictability drops off dramatically. For simulations in the gas phase, this drop-off arose from the fact that many of the trajectories would fail to reside in the product well that they initially accessed. In simulations of condensed-phase behavior, some of the same hopping between product wells could still be seen, but to a reduced extent. However, the bath atoms or molecules added a new measure of uncertainty, which was that the physics of how energy transfer from solute to bath occurred inevitably led to perturbation of the direction of trajectories across the PES. The more effective the bath was at suppressing the hopping between product wells, the more profound its effect on the initial directions of reactive trajectories.

It is relevant to note that, for the reactions studied here, there were substantial differences in PE between the two key saddle points: 35 kcal/mol in the case of cyclopropylidene and 25 kcal/mol in the case of TMCP. These features of the PES guaranteed that the products would be chemically activated by amounts far above the critical energies required for product interconversion. It is likely that reactions having smaller PE differences between the two corresponding saddle points would have less propensity to exhibit product interconversion, although whether it would be suppressed completely is hard to predict from one case to another.

The study of Ess and co-workers on the dimerization of cyclopentadienone and the formation of semibullvalene by N_2_ extrusion^23^ can profitably be compared with the present work. They found ML models that could predict with high success which product well would initially be accessed. Interestingly, their best model was based on the technique of boosting,^58^ as was also found in the present work, although the specific algorithms were different in the two studies. In the case of the cyclopentadienone dimerization, Ess and co-workers ran their trajectories for 400 fs, during which time none of the trajectories exhibited hopping between product wells. It is unclear whether such behavior would have been observed had the trajectories been run for longer. For the N_2_ extrusion reaction, they did observe product-well hopping during the 400 fs runs, and, just as here, found that it resulted in severely degraded predictive ability. It should also be noted that Ess and co-workers took the trouble to analyze which particular atomic motions were principally responsible for the success of their predictive models. Such analyses have not been undertaken in the present work.

For simulations including bath atoms or molecules in the present work, the predictive models were identical to those developed for the gas-phase reactions. One might reasonably question whether one could not have had better success by training models on data generated from condensed-phase simulations. However, further thought suggests that this would not be an effective strategy. If a particular bath had no effect on the directions of the trajectories (compared to identical initial conditions in the gas phase), then success in predictions for that bath and for the gas phase would necessarily be identical. On the other hand, if the bath did perturb the directions of the trajectories, then improving the predictive capability for the condensed-phase simulations could only be achieved by including at least some data on the positions and/or momenta of the bath atoms. However, then one faces a daunting task of generating sufficient data for the training to be effective. The unstructured nature of liquids would seem to make this extremely difficult. Given that ML has apparently solved the protein folding problem,^59,60^ one hesitates to say that it could not achieve high predictive capability for condensed-phase dynamics, but for now it looks like a very challenging task.

## Supporting Information Available

Details of electronic structure calculations; proof of the translational and rotational invariance of the chirality metric; calculation of the optimum LST approximation to the IRC for cyclopropylidene ring opening; details of the artificial neural networks used in the calculation of energies and derivatives for cyclopropylidene and TMCP; and details of the boosted tree machine learning model for predicting the initially accessed minima of TMCP ring-opening trajectories (PDF)
