# Supplementary material for: Prediction of Kinetic Product Ratios: Investigation of a Dynamically Controlled Case
Source: J Phys Chem A. 2023 Jan 3;127(1):224–39. doi: 10.1021/acs.jpca.2c08301 (PMC9841574; doi:10.1021/acs.jpca.2c08301)
Supplement: Supplementary file 1 — jp2c08301_si_001.pdf [file jp2c08301_si_001.pdf]

Prediction of Kinetic Product Ratios: Investigation of a Dynamically Controlled Case.

Barry K. Carpenter*
School of Chemistry, Cardiff University, Main Building,
Park Place, Cardiff CF10 3AT, United Kingdom. Email: carpenterb1@cardiff.ac.uk.

Supporting Information

Table of Contents

S1. Details of Electronic Structure Calculations — Pp. S01 – S04
S2. Proof of the Translational and Rotational Invariance of the Chiral Metric — Pp. S04 – S05
S3. Calculation of the Optimum LST Approximation to the IRC for Cyclopropylidene Ring Opening — Pp. S05 – S07
S4. Details of the Artificial Neural Networks Used in the Calculation of Energies and Derivatives for Cyclopropylidene and TMCP — Pp. S07 – S10
S5. Details of the Boosted Tree Machine Learning Model for Predicting the Initially Accessed Minima of TMCP Ring-Opening Trajectories — Pp. S10 – S11
S6. References — Pp. S11 – S12

S1. Details of Electronic Structure Calculations

Electronic structure calculations were carried out with the Gaussian 09[1] (for the DFT model) and GAMESS-US[2] (for the CASSCF model) programs. The following stationary points were located:

Cyclopropylidene (CASSCF(4,4)/6-311G(3d,2p)):

```
C    -0.75214064     0.00000000     0.36323583
C     0.00000000     0.00000000    -0.92689455
C     0.75214064     0.00000000     0.36323583
H    -1.28624117     0.90351367     0.59580702
H    -1.28624117    -0.90351367     0.59580702
H     1.28624117     0.90351367     0.59580702
H     1.28624117    -0.90351367     0.59580702
  E = -115.8428411220 hartree
ZPE =    0.056937 hartree (0 imaginary frequencies)
```

Cyclopropylidene Ring-Opening Saddle Point (CASSCF(4,4)/6-311G(3d,2p)):

```
C    -0.25507653    -0.01473413     0.98224562
C     0.72730911     0.08925166     0.00000000
C    -0.25507653    -0.01473413    -0.98224562
H    -1.28808260     0.33594185     0.98146909
H    -0.00301877    -0.69138402     1.83642673
H    -1.28808260     0.33594185    -0.98146909
H    -0.00301877    -0.69138402    -1.83642673
  E = -115.9084117040 hartree
ZPE =    0.052446 hartree (1 imaginary frequency: 104.63i cm-1)
```

Cyclopropylidene Ring-Opening Saddle Point (UωB97X-D/cc-pVTZ):

C	-0.21751532	-0.01936705	1.08810794
C	0.63075870	0.07001179	0.00000000
C	-0.21751532	-0.01936705	-1.08810794
H	-1.29401886	0.20249045	1.04652202
H	0.13031825	-0.38845199	2.05135894
H	-1.29401886	0.20249045	-1.04652202
H	0.13031825	-0.38845199	-2.05135894

E = -116.529202760 hartree
ZPE = 0.068820 hartree (1 imaginary frequency: 382.49i cm^{-1})

Allene (CASSCF(4,4)/6-311G(3d,2p)):

C	0.00000000	0.00000000	1.31095779
C	0.00000000	0.00000000	0.00000000
C	0.00000000	0.00000000	-1.31095779
H	-0.65023845	-0.65023845	1.86388254
H	0.65023845	0.65023845	1.86388254
H	0.65023845	-0.65023845	-1.86388254
H	-0.65023845	0.65023845	-1.86388254

E = -115.9508636367 hartree
ZPE = 0.056275 hartree (0 imaginary frequencies)

Allene Internal Rotation Saddle Point (CASSCF(4,4)/6-311G(3d,2p)):

C	-1.27076852	0.00000000	-0.13466822
C	0.00000000	0.00000000	0.38603163
C	1.27076852	0.00000000	-0.13466822
H	-2.13089275	0.00000000	0.50354284
H	-1.43285263	0.00000000	-1.19734299
H	2.13089275	0.00000000	0.50354284
H	1.43285263	0.00000000	-1.19734299

E = -115.8791722143 hartree
ZPE = 0.053233 hartree (1 imaginary frequency: 1454.1i cm^{-1})

TMCP (UB3LYP-D3/6-31G(d,p):

C	0.74768502	-0.04916700	0.06558000
C	-0.00000000	-0.00000500	1.37459099
C	-0.74768400	0.04916700	0.06558300
C	-1.51421499	-1.27856398	-0.01850400
C	-1.54604697	1.24078703	-0.43217599
C	1.54605103	-1.24078703	-0.43217701
C	1.51420903	1.27856696	-0.01850200
H	-1.08694804	-2.06299996	0.61609399
H	-1.51497996	-1.65170705	-1.04928100
H	-2.54633594	-1.12467802	0.30886301
H	-0.96818602	2.16686010	-0.40852600
H	-2.43917608	1.38569999	0.18620799
H	-1.87160003	1.07727504	-1.46654701
H	0.96817797	-2.16685295	-0.40856901
H	2.43915701	-1.38572395	0.18623599
H	1.87164295	-1.07725596	-1.46653199

H	1.08696902	2.06298089	0.61614197
H	1.51492798	1.65174401	-1.04926598
H	2.54634595	1.12467301	0.30881399

E = -273.841238166 hartree
ZPE = 0.167518 hartree (0 imaginary frequencies)

TMCP Ring Opening Saddle Point (UB3LYP-D3/6-31G(d,p):

C	0.04061583	0.18401755	-0.89091897
C	0.57483965	1.18152630	0.00000000
C	0.04061483	0.18401770	0.89091909
C	1.11407101	-0.41893846	1.79452896
C	-1.35843718	-0.15143199	1.39052999
C	1.11407304	-0.41894174	-1.79452598
C	-1.35843503	-0.15142825	-1.39053297
H	1.06265175	-1.51507986	1.76898313
H	0.95771253	-0.10387140	2.83454895
H	2.11141014	-0.10721608	1.48163700
H	-2.13446069	0.50156748	0.99659503
H	-1.34461737	-0.01272492	2.47709894
H	-1.64763784	-1.19332230	1.20573711
H	1.06265080	-1.51508307	-1.76898086
H	2.11141181	-0.10722333	-1.48163104
H	0.95771956	-0.10387296	-2.83454704
H	-2.13445759	0.50157326	-0.99659801
H	-1.64763892	-1.19331837	-1.20574093
H	-1.34461319	-0.01272137	-2.47710109

E = -273.825643955 hartree
ZPE = 0.166427 hartree (1 imaginary frequency: 266.83i cm^{-1})

Tetramethylallene (UB3LYP-D3/6-31G(d,p):

C	0.00000000	0.00000000	1.31187105
C	0.00000000	0.00000000	-1.31187105
C	0.00000000	0.00000000	0.00000000
C	-1.28428698	0.00000000	-2.11510611
C	-0.00000000	1.28428698	2.11510611
C	-0.00000000	-1.28428698	2.11510611
C	1.28428698	0.00000000	-2.11510611
H	-1.33156300	-0.88134599	-2.76805902
H	-0.88134599	1.33156300	2.76805902
H	0.88134599	-1.33156300	2.76805902
H	1.33156300	0.88134599	-2.76805902
H	-1.33156300	0.88134599	-2.76805902
H	0.88134599	1.33156300	2.76805902
H	-0.88134599	-1.33156300	2.76805902
H	1.33156300	-0.88134599	-2.76805902
H	-2.16616607	0.00000000	-1.47074902
H	0.00000000	2.16616607	1.47074902
H	-0.00000000	-2.16616607	1.47074902
H	2.16616607	0.00000000	-1.47074902

E = -273.957395527 hartree
ZPE = 0.1688825 hartree (0 imaginary frequencies)

Tetramethylallene Internal Rotation Saddle Point (UB3LYP-D3/6-31G(d,p):

```
C      1.29565501      0.00001898      0.08948229
C      0.00000000      0.00000000      0.51872128
C     -1.29565501     -0.00001896      0.08948226
C     -1.77616704     -0.00001056     -1.35038376
C     -2.42182112      0.00001192      1.09605134
C      1.77616704      0.00001062     -1.35038376
C      2.42182112     -0.00001193      1.09605134
H     -2.40897274     -0.87904501     -1.53977966
H     -0.96776903     -0.00051619     -2.07955480
H     -2.40811825      0.87958491     -1.54004872
H     -3.06898260     -0.87931854      0.95922029
H     -3.06905818      0.87926543      0.95908427
H     -2.03839803      0.00010760      2.11725521
H      0.96776903      0.00051627     -2.07955480
H      2.40811729     -0.87958491     -1.54004872
H      2.40897369      0.87904412     -1.53977966
H      3.06898260      0.87931854      0.95922029
H      3.06905818     -0.87926543      0.95908427
H      2.03839803     -0.00010764      2.11725426
  E = -273.850629607 hartree
ZPE =    0.164823 hartree (1 imaginary frequency: 80.08i cm-1)
```

S2. Proof of the Translational and Rotational Invariance of the Chiral Metric

In the main text, the assertion is made that the determinant χ is translationally and rotationally invariant.

$$\chi = \begin{vmatrix} x_1 & y_1 & z_1 & 1 \\ x_2 & y_2 & z_2 & 1 \\ x_3 & y_3 & z_3 & 1 \\ x_4 & y_4 & z_4 & 1 \end{vmatrix} \tag{S1}$$

The proof of translational invariance is straightforward. It is a general property of determinants that adding one column, multiplied by an arbitrary scalar, to another leaves its value unchanged. Because the fourth column of χ consists of 1s, that means that adding an arbitrary scalar to each element of any column leaves its value unchanged. However, such an addition corresponds to translating the structure along the axis specified by the chosen column.

Proof of rotational invariance is less straightforward. It was accomplished by using the Mathematica symbolic algebra program.[3] The steps are shown below.

Step 1, define a 3x4 matrix, **q**, for the Cartesian Coordinates of four atoms:

q = {{x_1,x_2,x_3,x_4},{y_1,y_2,y_3,y_4},{z_1,z_2,z_3,z_4}};

Step 2, define a 3D rotation matrix for rotation by angle ϑ about axis (x_0,y_0,z_0):

rotM = RotationMatrix[ϑ,{x_0,y_0,z_0}];

Step 3, premultiply the Cartesian coordinate matrix by the rotation matrix:

rq = Dot[**rotM**,**q**];

Step 4, assemble the determinant χ from the transpose of **rq** plus a column of 1s.

χ={{**rq**[[1,1]],**rq**[[2,1]],**rq**[[3,1]],1},{**rq**[[1,2]],**rq**[[2,2]],**rq**[[3,2]],1},{**rq**[[1,3]],**rq**[[2,3]],**rq**[[3,3]],1},{**rq**[[1,4]],**rq**[[2,4]],**rq**[[3,4]],1}};

Step 5, expand the determinant χ in the domain of real numbers:

Simplify[Det[χ],{x_0∈Reals,y_0∈Reals,z_0∈Reals}]

Result:

$$x_2 y_3 z_1 - x_2 y_4 z_1 - x_1 y_3 z_2 + x_1 y_4 z_2 - x_2 y_1 z_3 + x_1 y_2 z_3 - x_1 y_4 z_3 + x_2 y_4 z_3 + x_4 (y_2 z_1 - y_3 z_1 - y_1 z_2 + y_3 z_2 + y_1 z_3 - y_2 z_3) + x_2 y_1 z_4 - x_1 y_2 z_4 + x_1 y_3 z_4 - x_2 y_3 z_4 + x_3 (y_4 z_1 + y_1 z_2 - y_4 z_2 - y_1 z_4 + y_2 (-z_1 + z_4))$$

One finds that ϑ, x_0, y_0, and z_0 are absent from the result, thereby demonstrating that the determinant of χ is invariant with respect to arbitrary rotation.

S3. Calculation of the Optimum LST Approximation to the IRC for Cyclopropylidene Ring Opening

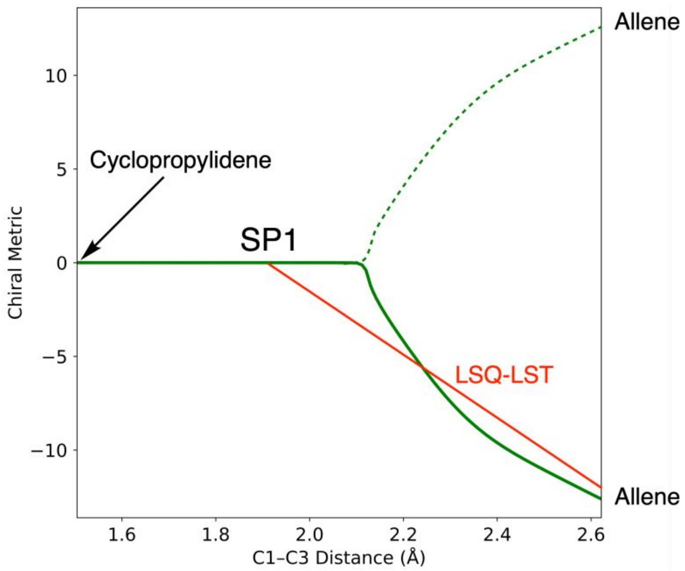

Figure S1. Projection of the cyclopropylidene ring-opening IRC into the 2D space used for trajectory analysis. The calculated IRC is the solid green curve. The dashed green curve is the second branch of the bifurcating IRC, inferred by symmetry. The red line, labeled LSQ-LST, is a schematic representation of the optimum LST approximation to the segment of IRC from SP1 to a product.

As described in section 3.1.1 of the main text, one of the methods tested for prediction of initially selected products from trajectory initial conditions involved the generation of a linear-synchronous-transit (LST) approximation to the IRC. For convenience, Fig. 6 from the main text is reproduced here as Fig. S1.

In reality, calculation of the LSQ-LST was carried out in the full-dimensional, mass-weighted Cartesian coordinates, rather than the 2D projection of Fig. SX1. This was accomplished as follows. Point *i* of the LSQ-LST was represented as:

$$\mathrm{LSQ\text{–}LST_i} = \mathrm{IRC_0} + i \times \mathrm{c} \quad \text{(S2)}$$

Here $\mathrm{IRC_0}$ is the structure of saddle point **SP1** in mass-weighted Cartesian coordinates and expressed as a 1D vector. The vector c consists of coefficients whose values are determined by minimizing the function:

$$\sum_i^M \sum_j^{3N} (\mathrm{IRC}_{ij} - \mathrm{LSQ\text{–}LST}_{ij})^2 \quad \text{(S3)}$$

Where index *i* specifies which point of the IRC one is considering and index *j* specifies which coordinate of that point one is considering. Consequently, *M* is the number of points in the IRC, and *N* is the number of atoms in the molecule.

The 2D representation in Fig. S1 indicates that the distance between the true IRC and its LST approximation must be zero by construction at the geometry of **SP1**, but there should be a second point where the two cross. That turns out to be very nearly true for the full-dimensional analogs as well, as indicated in Fig. S2, which depicts the squared distance between the true IRC and its LST approximation for each point of the IRC.

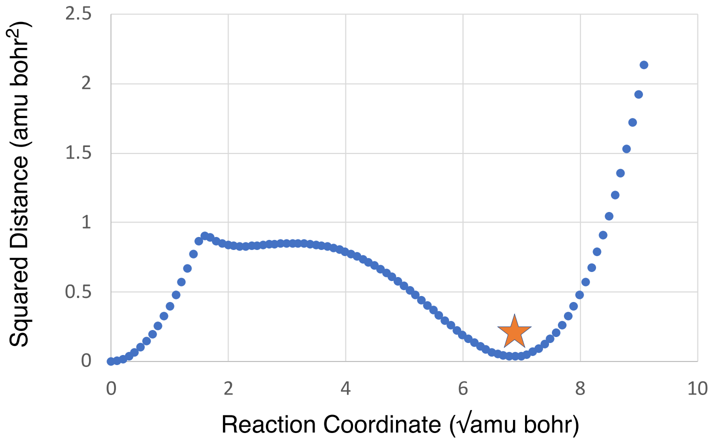

Figure S2. Squared distance (in mass-weighted coordinates) between the true IRC for cyclopropylidene ring opening and its optimum LST approximation. The red star indicates the reaction coordinate value at which the two almost cross.

The geometry corresponding to the near crossing point is depicted in Fig. S3, along with its pseudo-enantiomer, which would lead to the opposite product enantiomer.

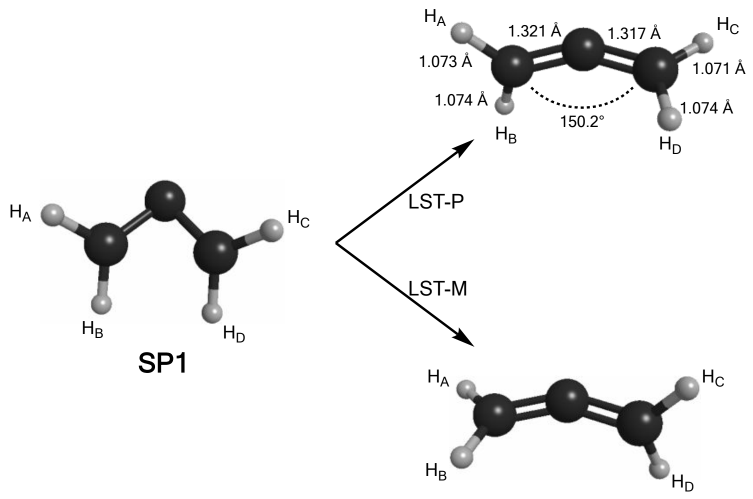

Figure S3. Geometry at which the optimum LST and true IRC almost cross (upper right). The structure without atom labeling is chiral. Its enantiomer becomes a pseudo-enantiomer once atom labels are introduced – i.e., with labels included, the two structures are actually diastereomeric.

In principle, there could be eight total paths from the SP1 saddle point to the two enantiomeric allenes, when atom labels are considered, even with the same atom connectivities maintained. However, trajectories were initiated by sampling around one particular diastereomer of the labeled SP1, and the sampling procedure did not allow generation of the other diastereomeric structures, and so only one LST need be generated for each product enantiomer.

With the LSQ-LSTs established, the procedure for making a product prediction from initial conditions of a given trajectory was as follows. The starting structure was referred to its center of mass as origin, and then rotated in mass-weighted coordinates for maximum overlap with the orientation of SP1 used to generate the LSQ-LSTs. This was accomplished by using the Kabsch algorithm.[4] The rotation angles required to achieve maximum overlap were recorded and then applied to the mass-weighted Cartesian velocities. The resulting velocities were flattened into a 1D vector, which was normalized. Finally, the dot product of this vector was taken with the unit vectors pointing along the directions of the two LSQ_LSTs. The larger value of the dot product indicated the preferred product.

S4. Details of the Artificial Neural Networks Used in the Calculation of Energies and Derivatives for Cyclopropylidene and TMCP

The MM-ANN method described in the main text made use of feedforward artificial neural networks (ANNs), the details of which came directly from the work Artrith, Urban, and Ceder (AUC).[5] The incorporation of the ANNs into the TINKER molecular mechanics package was based on the work of Chen *et al.*,[6] with differences noted below.

A feedforward ANN can be represented by the recurrence equation S4:

$$\mathbf{v}^{l} = f_{a}^{l}\left(\mathbf{w}^{l}\bullet\mathbf{v}^{l-1} + \mathbf{b}^{l}\right) \qquad \text{(S4)}$$

where $\mathbf{v}^l$ is the vector of values assigned to the neurons in the lth layer of the network. On the right-hand side of S4, f_a^l is the activation function for layer l, while $\mathbf{w}^l$ and $\mathbf{b}^l$ are vectors of weights and biases for the layer, whose values will be optimized during the training of the network. The values of $\mathbf{v}^{-1}$ are all set to unity, so that the input layer of the ANN, $\mathbf{v}^0$, does not depend on a prior layer. The input layer accepts structural information about the molecule for which energies and derivatives are required. However, the ANN model must return values that are independent of translation and rotation of the molecule, and also independent of permutations among chemically equivalent atoms. These requirements rule out conventional Cartesian or internal coordinates as inputs to the ANN. The AUC solution to this problem draws on an idea originally proposed by Behler and Parrinello, which is to define different atom types within a molecule and to train separate ANNs for each type. The overall energy is then simply the sum of the atomic contributions.

This approach can be recognized to be similar in philosophy to Benson's group-additivity method for calculation of heats of formation for molecules.[7] Benson tabulated a large number of enthalpy contributions for various atom groups. A group would be defined by a central atom plus all the atoms to which it was directly connected. The heat of formation for a molecule could then be calculated by identifying all its groups and then simply adding up the contributions from each. Often, some additional correction factors would be required to capture features such as ring strain, which could not be embedded in the group contributions, but with these corrections included, final heats of formation within ±1 kcal mol of the experimental value were typical for "normal" organic molecules.[7]

The AUC method expresses the environment of a particular atom type in terms of radial distribution functions (RDFs), which capture pairwise interactions between atoms and angular distribution functions (ADFs), which capture three-body interactions. The RDFs and ADFs are themselves expanded in a basis set of Chebyshev polynomials:

$$RDF_{t_i} = \sum_{\alpha}^{\alpha_{max}} \sum_{j}^{N} \sum_{k \neq j}^{N} T_{\alpha}\left(\frac{2r_{jk}}{R_c} - 1\right) f_c(r_{jk}) \tag{S5}$$

In eq. S5, t specifies the list of atom types, and t_i is the ith member of that list. T_α is the Chebyshev polynomial of order α. The limit α_{max} is the highest order of polynomial at which the expansion is truncated. N is the total number of atoms in the structure, r_{jk} is the distance between atoms j and k, R_c is the cutoff distance chosen for the expansion, and $f_c(r_{jk})$ is a cutoff function, defined in eq. S7. Chebyshev polynomials are defined by the recurrence relationship in eq. S6, with $T_0(x) = 1$ and $T_1(x) = x$.

$$T_{\alpha+1}(x) = 2xT_a(x) - T_{a-1}(x) \tag{S6}$$

$$f_c(r_{jk}) = \begin{cases} \left[\cos\left(\frac{\pi r_{jk}}{R_c}\right) + 1\right]/2 & (r_{jk} \leq R_c) \\ 0 & (r_{jk} > R_c) \end{cases} \tag{S7}$$

The ADFs are defined by eq. S8:

$$ADF_{t_i} = \sum_{\alpha}^{\alpha_{max}} \sum_{j}^{N} \sum_{k \neq j}^{N} \sum_{l \neq j, l \neq k}^{N} T_{\alpha}(\cos\theta_{kjl}) f_c(r_{jk}) f_c(r_{jl}) \qquad \text{(S8)}$$

where θ_{kjl} is the angle made by atoms *k* and *l* at atom *j*.

In the original work, training of the ANNs involved minimizing the cost function *C* (eq. S9):

$$C(\mathbf{w},\mathbf{b}) = \frac{1}{2}\sum_{i=1}^{N_{data}} \left[E_i^{ANN}(\mathbf{w},\mathbf{b}) - E_i^{QM}\right]^2 \qquad \text{(S9)}$$

where $E_i^{ANN}(\mathbf{w},\mathbf{b})$ is the total energy of the ith structure returned by all the relevant atomic ANNs, meaning that **w** and **b** in eq. S9 are vectors of weights and biases that encompass all layers of all atomic ANNs. N_{data} is the total number of structures for which one has calculated energies and derivatives using the chosen electronic structure method. E_i^{QM} is the reference quantum-mechanical energy for the ith structure. In the present work, the original cost function *C* was replaced by the new cost function *F*, defined in eq. S10.

$$F(\mathbf{w},\mathbf{b}) = \frac{1}{2}\sum_{i=1}^{N_{data}} \left[E_i^{ANN}(\mathbf{w},\mathbf{b}) - E_i^{QM} + E_i^{MM3}\right]^2 \qquad \text{(S10)}$$

Here, E_i^{MM3} is the energy of the ith structure at the MM3 molecular-mechanics level. The ANNs were thus trained to provide corrections to the MM3 energies and gradients. Minimization of *F* with respect to **w** and **b** was carried out by the limited memory Broyden-Fletcher-Goldfarb-Shanno algorithm, as in the original work.

All the calculations implied by eqs. S5-S10 were carried out with the free and open-source packages ænet and ænet-tinker.[5]

For the cyclopropylidene MM-ANN potential, three atom types were identified: C, Cq, and H, as illustrated below:

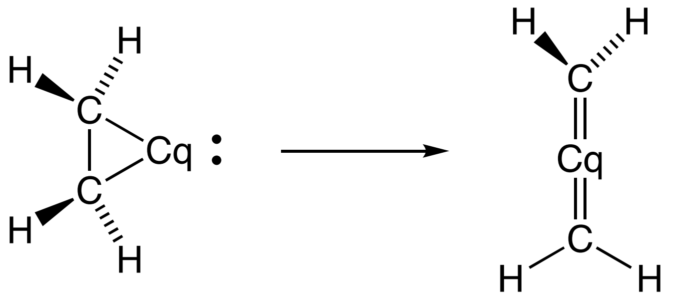

Three atomic ANNs were thus required, called C.ann, Cq.ann, and H.ann, respectively. All three ANNs had the following parameters:

Number of layers: 4
Number of nodes (without bias) and activation type per layer:
1 : 74
2 : 50 hyperbolic tangent (tanh)
3 : 50 hyperbolic tangent (tanh)
4 : 1 linear function (linear)

Minimal distance: 0.75 Å
Maximal cut-off: 5.0 Å
Radial *Rc*: 5.0 Å
Angular *Rc*: 4.0 Å
Radial order α_{max}: 25
Angular order α_{max}: 10

C.ann and H.ann both had all three atom types in their environments, whereas Cq.ann only had atom types C and H in its environment.

For the TMCP MM-ANN potential, four atom types were identified, as illustrated below:

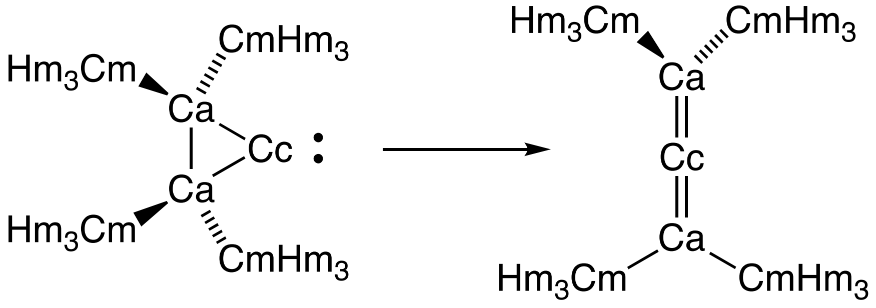

The four atomic ANNs: Ca.ann, Cc.ann, Cm.ann, and Hm.ann each had the same parameters listed above for the cyclopropylidene ANNs. Each of the ANNs had all four atom types in its environment, except for Cc.ann, which only had atom types Ca, Cm, and Hm in its environment.

In order to carry out the trajectory calculations in the presence of bath atoms or molecules, it was necessary to create dummy ANNs for each of the bath atom types. Even though the intent was to evaluate bath energy terms and bath-solute interaction terms purely by MM3 molecular mechanics, the ænet-tinker code expected to find an ANN for every atom type present. The dummy ANNs were created by training against data sets in which bath atoms were randomly located within their periodic-boundary box, with each of the random configurations being given a correction energy near zero. The energies could not be set exactly to zero because an internal rescaling within the code would overflow if that was attempted, but energies of $\pm 10^{-7}$ kcal/mol were accepted.

S5. Details of the Boosted Tree Machine Learning Model for Predicting the Initially Accessed Minima of TMCP Ring-Opening Trajectories

As described in the main text, Cartesian coordinates and conjugate momenta of the initial structures were converted to internal-coordinate equivalents by computing the Wilson B matrix, whose elements are $\frac{\partial \mathbf{q}}{\partial \mathbf{x}}$, where $\mathbf{q}$ is the vector of internal coordinates and $\mathbf{x}$ is the vector of Cartesian coordinates. This was accomplished in Python, using the autograd module (https://github.com/HIPS/autograd/blob/master/docs/tutorial.md, accessed 12/15/2022) to compute the co-ordinate derivatives. The trajectories were run for 150 fs, using the MM-ANN potential for TMCP. At the end of each trajectory, the result was classified as M, P, or R for, respectively, a product with a negative value of the chirality metric, a product with a positive value of the chirality metric or a recrossing trajectory that returned to the reactant. The initial

conditions and outcomes were tabulated for 1246 initial conditions, sampled from a conical ensemble at 298 K in the vicinity of the ring-opening saddle point. From these data, 1000 points were used for training the ML model and 246 for testing it.

The data were evaluated with the Apple "Create ML" software, run on a Macintosh Mini with the Apple M1 processor. The best results were obtained using a boosted tree model having the following characteristics:

Maximum Iterations:	25
Maximum depth:	10
Minimum Loss Reduction:	0
Minimum Child Weight:	0.1
Row Subsample Ratio:	1.0
Column Subsample Ratio:	1.0
Step Size:	0.3

It was able to reproduce trajectory outcomes with 99% accuracy for the training set and to predict outcomes with 98% accuracy for the test set.

Once trained, the model could be saved and used to predict new trajectory outcomes using the Python module coremltools (https://github.com/apple/coremltools, accessed 12/15/2022).

S6. References

1. Gaussian 09, Revision D.01: Frisch, M. J.; Trucks, G. W.; Schlegel, H. B.; Scuseria, G. E.; Robb, M. A.; Cheeseman, J. R.; Scalmani, G.; Barone, V.; Mennucci, B.; Petersson, G.; et al. Gaussian, Inc., Wallingford CT, 2013.
2. Schmidt, M. W.; Baldridge, K. K.; Boatz, J. A.; Elbert, S. T.; Gordon, M. S.; Jensen, J. H.; Koseki, S.; Matsunaga, N.; Nguyen, K. A.; Su, S.; et al. General Atomic and Molecular Electronic Sructure System. *J. Comput. Chem.* **1993**, *14*, 1347-1363.
3. Wolfram Research, Inc., Mathematica, Version 12.1, Champaign, IL (2020).
4. Kabsch, W. A solution for the best rotation to relate two sets of vectors. *Acta Cryst*. **1976**, *A32*, 922.
5. Artrith, N.; Urban, A.; Ceder, G. Efficient and accurate machine-learning interpolation of atomic energies in compositions with many species. *Phys. Rev. B* **2017**, *96*, Article 014112.
6. Chen, M. S.; Morawietz, T.; Mori, H.; Markland, T. E.; Artrith, N. AENET-LAMMPS and AENET-TINKER: Interfaces for accurate and efficient molecular dynamics simulations with machine learning potentials. *J. Chem. Phys.* **2021**, *155*, Article 0063880.
7. Benson, S. W. *Thermochemical Kinetics*, 2nd Ed. Wiley, New York, 1976.
